# Not that young: combining plastid phylogenomic, plate tectonic and fossil evidence indicates a Palaeogene diversification of Cycadaceae

**DOI:** 10.1093/aob/mcab118

**Published:** 2021-09-14

**Authors:** Jian Liu, Anders J Lindstrom, Thomas E Marler, Xun Gong

**Affiliations:** 1 CAS Key Laboratory for Plant Diversity and Biogeography of East Asia, Kunming Institute of Botany, Chinese Academy of Sciences, Kunming 650201, Yunnan, China; 2 Department of Economic Plants and Biotechnology, Yunnan Key Laboratory for Wild Plant Resources, Kunming Institute of Botany, Chinese Academy of Sciences, Kunming 650201, Yunnan, China; 3 Global Biodiversity Conservancy, 144/124 Moo3, Soi Bua Thong, Bangsalae, Sattahip, Chonburi 20250, Thailand; 4 Western Pacific Tropical Research Center, University of Guam, UOG Station, Mangilao, GU 96923, USA; 5 University of Chinese Academy of Sciences, Beijing 100049, China

**Keywords:** Cycadaceae, Cycads, origin, Palawan, Palaeogene, plastid phylogenomics

## Abstract

**Background and Aims:**

Previous molecular dating studies revealed historical mass extinctions and recent radiations of extant cycads, but debates still exist between palaeobotanists and evolutionary biologists regarding the origin and evolution of Cycadaceae.

**Methods:**

Using whole plastomic data, we revisited the phylogeny of this family and found the Palawan endemic *Cycas* clade was strongly related to all lineages from Southeast Eurasia, coinciding with a plate drift event occurring in the Early Oligocene. By integrating fossil and biogeographical calibrations as well as molecular data from protein-coding genes, we established different calibration schemes and tested competing evolutionary timelines of Cycadaceae.

**Key Results:**

We found recent dispersal cannot explain the distribution of Palawan *Cycas*, yet the scenario including the tectonic calibration yielded a mean crown age of extant Cycadaceae of ~69–43 million years ago by different tree priors, consistent with multiple Palaeogene fossils assigned to this family. Biogeographical analyses incorporating fossil distributions revealed East Asia as the ancestral area of Cycadaceae.

**Conclusions:**

Our findings challenge the previously proposed Middle–Late Miocene diversification of cycads and an Indochina origin for Cycadaceae and highlight the importance of combining phylogenetic clades, tectonic events and fossils for rebuilding the evolutionary history of lineages that have undergone massive extinctions.

## Introduction

Establishing the evolutionary timescale for the tree of life is a central yet elusive goal of evolutionary biology (De Baets *et al.*, 2016). The most common approach in molecular dating to calibrate a node relies on known fossil information. However, accurately dating lineages lacking appreciable or agreed fossil records remains challenging. Among alternative approaches additional to fossils or stratigraphy (Bromham and Penny, 2003; Hipsley and Müller, 2014), a less direct method of calibration can be used when evolutionary divergence events can be associated with geological or climatic events of known age, i.e. biogeographical/tectonic calibration (Heads, 2012, 2019). The close tectonic and palaeogeological correlations between extant clades, which spatially coincide with the nodes, provide an obvious approach for calibrating and dating clades (Ho *et al.*, 2015; De Baets *et al.*, 2016; Heads, 2019). The advantage of using these biogeographical events is that they are well dated, and can give more robust and accurate estimations than using single fossil calibration (Landis, 2017; Gutiérrez-Ortega *et al*., 2018). However, biogeographical calibrations need to justify several key assumptions: that the biogeographical event has had measurable evolutionary and genetic impacts (i.e. geological or climatic events are assumed to have affected the branching process), and that the event can be dated independently (i.e. the age of the biogeographical event is known) (Ho *et al.*, 2015). Despite multiple concerns (e.g. strong pre-assumption, uncertain ages of such events) in applying calibrations based on geological or climatic events (Forest, 2009; Kodandaramaiah, 2011; Hipsley and Müller, 2014), simulations indicate biogeographical dating performs well whenever palaeogeography imposes a constraint on biogeographical character evolution (Landis, 2017). Thus, biogeographical calibrations have the potential to make a valuable contribution to molecular dating if implemented and interpreted carefully.

Cycads have long been considered as the most ancient living seed plant lineage (Brenner *et al.*, 2003). Among cycads, one of the most compelling and contentiously debated questions is the origin and evolution of the family Cycadaceae. Containing only one genus (*Cycas* L.), Cycadaceae is the most diversified and extensively distributed group in extant cycads and is the sister to all other cycads (Hill, 2008; Salas-Leiva *et al.*, 2013; Liu *et al.*, 2018). Recent molecular dating for living Cycadaceae proposed their post-Neogene diversification (Nagalingum *et al.*, 2011; Salas-Leiva *et al.*, 2013; Condamine *et al.*, 2015). However, this was questioned by palaeobotanists (Su *et al.*, 2014) who implied a much earlier diversification of *Cycas* by arguing that multiple Palaeogene cycad fossils (Yokoyama, 1911; Krassilov, 1978; Su *et al.*, 2014) can be assigned to the extant *Cycas* because of the single-vein midrib which is unique to this group (Hermsen *et al.*, 2007). In addition, biogeographical analyses for Cycadaceae also revealed inconsistent evolutionary histories and conflicting ancestral areas (South China: Xiao and Möller, 2015; Indochina: Mankga *et al.*, 2020), raising the necessity to revisit the origin and differentiation of this family.

**Fig. 1.**
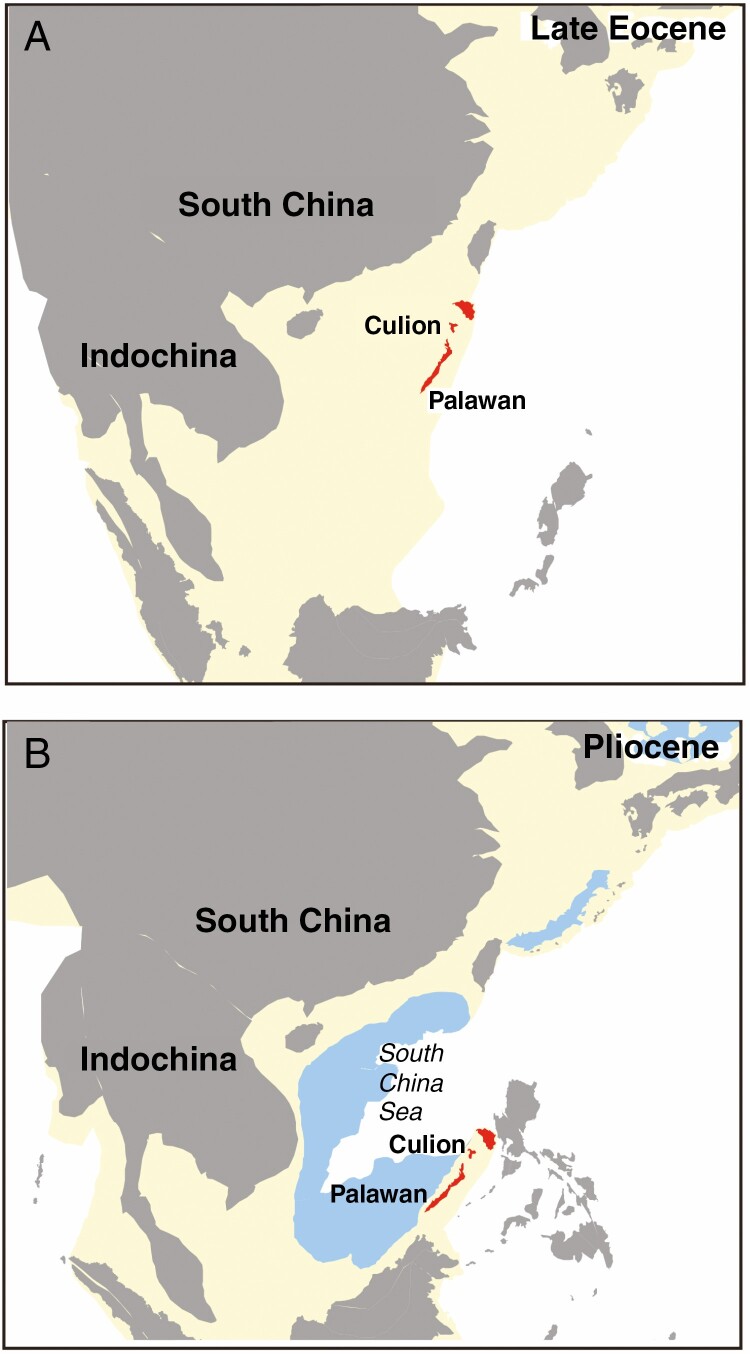
Illustration of the proposed location of the Palawan microcontinental block (red) in (A) the Early Eocene (35 Ma) and (B) Pliocene (5 Ma), based on reconstructions of the geological history of Southeast Asia (Hall, 2012). The white region denotes the deep sea. Pale yellow regions represent submarine continental margins. Blue regions indicate sea-floor spreading.

Within *Cycas*, section *Wadeae* is endemic to the closely associated islands of Culion and Palawan, belonging to the Palawan microcontinent that now forms part of the Philippine archipelago (Fig. 1). The rifting of North Palawan microcontinents from continental Eurasia since the Late Eocene has been well documented in many paleogeographical studies (Almasco *et al.*, 2000; Suzuki *et al.*, 2000; Yumul *et al.*, 2009; see also Fig. 1). Most terrestrial animals and plants, especially those with limited dispersal ability over ocean barriers, may have undergone vicariant speciation due to this process of plate movement, which gave rise to the ‘Palawan Arc’ hypothesis (Blackburn *et al.*, 2010). The species from section *Wadeae* morphologically closely resemble those of sections *Panzhihuaenses*, *Asiorientales* and *Stangerioides* found in continental East Asia or along margins of the shallow sea basin of East Asia, suggesting the analogous ‘rafting Palawan’ hypothesis for the distribution of *Wadeae* (Tang, 2004). Although Hill (1999) recognized section *Wadeae* as close to section *Stangerioides* by cladistic analysis based on morphological data, previous phylogenetic studies based on different molecular markers generated incongruent positions of this section in *Cycas* (Xiao and Möller, 2015; Liu *et al.*, 2018; Mankga *et al.*, 2020). The clear separation between *Cycas* from East Asia and Palawan provides a framework to test the utility of biogeographical evidence in the molecular dating of *Cycas* but has not been applied in any previous study involving the dating of this genus.

Plastid phylogenomics has facilitated progress in rebuilding the timescale for the tree of life over the last decade (Moore *et al*., 2010; Davis *et al.*, 2014; Li *et al.*, 2019) and has been widely applied to resolve deep relationships of particularly recalcitrant lineages, such as those that have undergone recent radiations (Ma *et al.*, 2014; Barrett *et al.*, 2016). In the present study, we first reconstruct the phylogenetic framework of Cycadaceae using whole plastomic data and link this phylogeny to the distribution of taxa. Then we combine palaeogeographical and fossil evidence to establish different calibration schemes, use molecular dating to estimate the ages of major phylogenetic nodes under different calibration schemes, and test their robustness among different effects. We then discuss which timeline based on calibration scenarios best explains the historical biogeography of Cycadaceae. By understanding the timescale and biogeography of extant *Cycas*, we aim to settle debates on the spatial and temporal origin of this group and further discuss how to better accommodate the conflicts between clock and rock using the biogeographical calibrations.

## MATERIALS AND METHODS

### Sampling and DNA extraction

We sampled 47 complete plastomes of Cycadaceae, covering all six sections and all major clades from a previous phylogeny with nearly complete sampling (Liu *et al.*, 2018), with 40 newly sequenced plastomes. Additionally, we added 11 published plastomes, including all other nine genera (Zamiaceae) from Cycadales, and two *Ginkgo* accessions to our dataset. Sampling information and accessions of all materials used in this study can be found in Supplementary Data Table S1. Total genomic DNA was extracted from silica gel-dried materials by a modified CTAB method (Doyle, 1991).

### Plastome sequencing, assembly and annotation

We used the genome skimming method (Dodsworth, 2015) to obtain the *Cycas* plastomes. A total of 2 Gb of sequencing data from the Illumina Hiseq Platform (Novogene, Beijing, China) were filtered for each sample and used for plastome assembly in the *get_organelle* pipeline (Jin *et al.*, 2020) by using *Cycas szechuanensis* (NC042668) as a reference. The resulting contigs were trimmed and further edited in Bandage v.0.7.1 (Wick *et al.*, 2015) to obtain the quadrantal structure contig. We applied both the PGA pipeline (Qu *et al.*, 2019) and GeSeq (Tillich *et al.*, 2017) to perform plastome annotation using *C. szechuanensis* as a reference. The annotations were compared, double-checked and adjusted in Geneious prime v.2020 (Kearse *et al.*, 2012).

### Phylogenetic analyses and divergence time estimation

Phylogenetic reconstructions were implemented in IQTREE v.2.1.1 (Minh *et al.*, 2020) to infer the maximum-likelihood (ML) tree using the ultrafast bootstrap approximation method (Hoang *et al.*, 2018) with 1000 replicates. We ran the phylogenetic analyses based on two datasets: protein-coding (PC) regions and the whole plastomic (WP) dataset.

Age estimation was conducted in the BEAST package v.2.6.1 (Bouckaert *et al.*, 2019) based on the PC dataset. We employed three fossil calibrations from a comprehensive dating analysis of all cycads based on six fossils (Condamine *et al.*, 2015). We did not incorporate all six fossils because our study focused only on Cycadacae, and the remaining three fossils were used as internal calibrations of Zamiaceae. These fossils, *Crossozamia chinensis* (Gao and Thomas, 1989), *Antarcticycas schopfii* (Hermsen *et al.*, 2006) and *Dioon inopinus* (Hollick, 1932), are the oldest known Cycadophyta fossils, the closest relatives to extant cycads (Martínez *et al.*, 2012), and the closest lineage to extant *Dioon*, respectively. Following Condamine *et al.* ( 2015), we applied these three fossils to constrain the stem (364.7–265.1 million years ago, Ma) and crown nodes (364.7–235.0 Ma) of Cycadales, and the crown of Zamiaceae (265.1–56 Ma) respectively, using the uniform prior distribution.

Our plastomic phylogeny strongly resolved the Palawan lineages as sisters to the extant lineages from South China (see Results), consistent with a vicariance event caused by tectonic movements (i.e. opening of the South China Sea; Tang, 2004). As there is no consensus on the precise time when the seafloor of the South China Sea started to spread (~37–32 Ma; Briais *et al.*, 1993; Hsu *et al.*, 2004) after Late Eocene to Early Oligocene, we applied a uniform prior distribution of 37–32 Ma as an internal biogeographical calibration for the split of lineages between Palawan and continental Eurasia based on the reconstruction of Cenozoic Southeast Asia (Hall, 2002), to compare its effects for age estimation.

To evaluate the influence of crown constraint in age estimation for clades of Cycadaceae, we also conducted a separate molecular dating for cross-validation by using the stratigraphic age of *Cycas fushunensis* fossils (47.5 ± 1 Ma) (Su *et al.*, 2014) as a minimum constraint to the crown of extant *Cycas*, and the fossil age of *A. schopfii* to constrain the upper bound, using a uniform prior distribution (i.e. 235.0–47.5 Ma), along with the three cycad fossils above, to estimate the internal node ages.

**Fig. 2.**
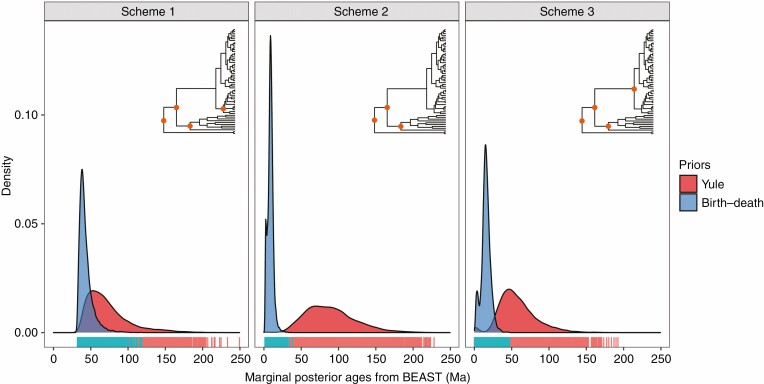
Density plots of marginal posterior age distributions of crown node *Cycas* based on three calibration schemes (Schemes 1–3) and different tree priors (birth–death and Yule). Ages were extracted from 10 000 posterior trees from BEAST analyses. The phylograms above the density plots show the calibrated nodes (orange) of each scheme as described in the Methods.

As a result, we set three different calibration schemes: (1) combined three-fossil and tectonic events (Palawan block-split event), (2) a three-fossil calibration (internal nodes within the *Cycas* phylogeny unconstrained), and (3) a four-fossil calibration scheme with *Cycas* crown constrained (Fig. 2, insets). In each calibration scheme, we compared the performance of both the Yule and birth–death priors, because the choice of the branching process prior can have a drastic influence on clade ages (Condamine *et al.*, 2015). We utilized a default uniform prior with a starting value of 1 for the Yule birth rate. For the birth–death prior, we also set the uniform prior as default from 0 to 10^4^ for the speciation rate and 0 to 1 for the relative extinction rate; starting values were 1.0 for speciation and 0.5 for relative extinction. We used the nested sampling (NS) method (Russel *et al.*, 2019) implemented in BEAST to choose which branching process prior was favoured for each calibration scenario. We then compared the estimated marginal posterior age distribution for major internal nodes by different schemes described above. After the run, we used the ‘birthdeath’ function implemented in the R package *ape* (Paradis and Schliep, 2018), which fits by ML of a birth–death model to the branching times, to compute the extinction rates of the obtained maximum clade credibility (MCC) consensus trees of each scheme based on the birth–death prior.

We also evaluated the effects of using different dataset partitions, subsamples of data, clock models and tree priors in estimating the timeline of *Cycas*. First, we created three datasets, one with all sites from the PC dataset included (nt123: 84 675 nucleotides), one with all third codon sites removed (nt12: 56 450 nucleotides) and another with only third codon sites retained (nt3: 28 225 nucleotides). This allowed us to examine the effect of saturation at third codon positions. The recommended substitution model in BEAST analyses for the partitioned dataset was determined by PartitionFinder2 (Lanfear *et al.*, 2017), all yielding GTR+G+I+X. We further used the PhyloMAd software (Duchene *et al*., 2018) to test the model adequacy and sequence saturation. Second, we drew a random subsample of 10, 20, 40 (Supplementary Data Table S2) and all 87 genes to examine how the date estimates responded to increases in the size of the dataset. Third, we used the PC dataset to examine the impacts of all four clock models available in BEAST: strict clock (SC), random local clock (RLC), and two uncorrelated relax clock models – exponential (RCE) and lognormal (RCL). We then used the NS method to choose the best-fitting clock model. Last, we also examined the effects of different tree priors (i.e. Yule and birth–death) as described above on internal age estimation. All the above analyses were run using calibration Scheme 1. We analysed the partitioned and subsampled data in BEAST using the RCL model to allow a range of optimal values for the among-lineage rate of substitution and using the birt–-death prior to allow extinction, as they were revealed as the best-fit models (Table 1; Table S3). For all the above analyses, we compared the mean posterior ages and their 95% highest posterior density (HPD) intervals for major internal nodes under different effects.

**Table 1.** Marginal likelihood estimates of different clock models inferred by the nested sampling method in BEAST. The favoured prior is in bold type as the likelihood is greater than any other by 2SD.

Clock model	Marginal likelihood			
	Strick clock (Mean SD = 2.52)	Uncorrelated relax clock exponential (mean SD = 2.81)	Uncorrelated relax clock lognormal (mean SD = 2.80)	Random local clock (mean SD = 2.87)
Replicate 1	−221 370.84	−220 012.67	−220 012.61	−220 136.08
Replicate 2	−221 376.71	−220 022.77	−220 007.23	−220 442.91
Mean	−221 373.78	−220 017.72	−220 009.92	−220 289.50

For each analysis, we ran one or two repeats of 500 million generations and sampled the log every 50 000 generations to ensure it reaches a posterior effective sample size (ESS > 200) in Tracer v.1.7 (Rambaut *et al.*, 2018). In total, 500 million to 1 billion iterations were run in BEAST for each analysis, which generated 10 000–20 000 trees by sampling the tree every 50 000 generations. The first 10–30% of trees were discarded as burn-in before generating the MCC consensus tree in TreeAnnotator (Bouckaert *et al.*, 2019). The consensus MCC tree with estimated ages was visualized in Figtree v.1.4.4 (Rambaut *et al.*, 2018).

### Ancestral area reconstruction

To examine the ancestral distribution of *Cycas*, we conducted ancestral area reconstruction. Combining the distribution of extant *Cycas* and its fossils, as well as past and current continental positions, we defined five biogeographical areas: A, East Asia, south to the Red River region; B, Indochina and India; C, Palawan and Culion islands; D, Southeast Asia islands including Australia; and E, Africa. The biogeographical history of Cycadaceae was inferred by RASP v.4 (Yu *et al.*, 2015) using the statistical dispersal–extinction–cladogenesis (S-DEC) model (Ree and Smith, 2008), by using 1000 posterior trees derived from the BEAST analysis based on Scheme 1, given the week support on two nodes in the phylogeny (see Results).

To estimate the effect of fossils in the reconstruction of the ancestral area, we compared the inferred biogeographical history of Cycadaceae (1) by integrating the fossil distribution of *C. fushunensis* as sister to all extant *Cycas*, and (2) based on extant taxa. For the S-DEC analysis, we constrained the dispersal between some biogeographical regions using an adjacency matrix (Supplementary Data Table S4) and set equal dispersal probabilities between other regions through time. We also set the baseline rates of dispersal and local extinction to be ‘estimated’. All possible area combinations, with a maximum of two simultaneous areas, were permitted in RASP, as no *Cycas* species occur in more than two biogeographical areas as defined here.

## RESULTS

### Phylogenetic reconstruction

The plastomic phylogeny inferred by ML generated slightly conflicting topologies for some clades based on different datasets, with higher bootstrap probabilities (BP) for most clades by the WP than PC dataset (Supplementary Data Fig. S1). Therefore, the following results focus primarily on the phylogeny based on the WP dataset. Four major clades were generated within *Cycas* (Fig. S2), and a long branch was found for *C. taitungensis*. Regardless of which dataset was applied, all results strongly supported that sect. *Wadeae* (including *C. wadei* and *C. aenigma*) was sister to the clade comprising sections *Stangerioides*, *Panzhihuaenses* and *Asiorientales* (Fig. S1, clade I in Fig. S2). Sections *Panzhihuaenses* (*C. panzhihuaensis*) and *Asiorientales* (*C. revoluta* and *C. taitungensis*) were resolved as sisters to some species from section *Stangerioides* but with weak support (Fig. S2). Section *Indosinenses* was resolved as sister to a subclade of section *Cycas* (*Cycas* II in Fig. S2).

### Molecular dating

**Fig. 3.**
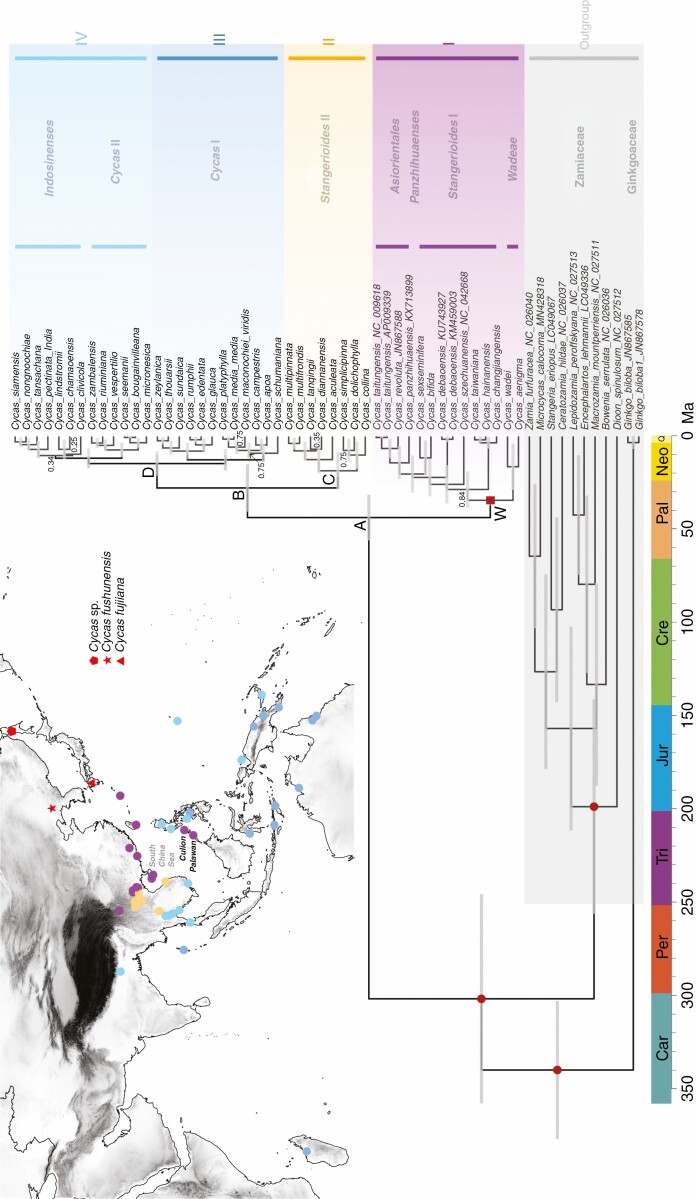
Chronogram of *Cycas* reconstructed by BEAST based on 87 protein-coding regions using the birth–death prior with Scheme 1 which combined fossil (red solid circles) and geological event (red solid square) calibrations. Morphologically recognized sections are annotated within the clades. Outgroups are shaded grey. Numbers at nodes denote the posterior probabilities (PP) of the clade as inferred by BEAST, with PP = 1 not indicated. Inset is the biogeographical pattern of *Cycas* with the distribution of the clades shown by different colours. Important geographical information and three fossils described as *Cycas* (Yokoyama, 1911; Krassilov, 1978; Su *et al.*, 2014) are indicated in the inset.

The MCC consensus tree generated by BEAST had an identical topology to the ML tree based on the WP dataset (Supplementary Data Fig. S3). However, different calibration schemes yielded contrasting node ages for the *Cycas* chronogram (Table 2, Fig. 2 and Fig. S4). Under the favoured birth–death prior (Table S3), the mean crown age of Cycadaceae was estimated as 42.88 Ma (95% HPD: 57.76–33.45 Ma, node A, Table 2, Fig. 3) for Scheme 1, and 9.33 Ma (95% HPD: 15.65–3.11 Ma) for Scheme 2 (Table 2; Fig. S5a). The mean crown ages of Cycadaceae were estimated to diverge in the Late Cretaceous (69.31 Ma, Scheme 1; 87.39 Ma, Scheme 2) based on the Yule prior (Table 2; Figs S5b and S6). In Scheme 3, the divergence times between Palawan and East Asian lineages (node W in Fig. 3) were 16.86 Ma (95% HPD: 26.37–8.63 Ma) and 56.58 Ma (95% HPD: 101.44–22.47 Ma) for birth–death and Yule priors, respectively (Table 2; Fig. S7). In general, Schemes 1 and 3 showed more age distribution overlaps between birth–death and Yule priors than Scheme 2 (Fig. 2; Fig. S4). For the MCC trees inferred by the birth–death prior, higher extinction rates were estimated from Scheme 2 (0.311) than Schemes 1 (0.097) and 3 (0.120).

**Table 2.** Summarized ages of main nodes/clades (labelled in Fig. 3) by different calibration schemes for Cycadaceae. Ranges in square brackets after the node age denote the 95% confidence interval of each node age. Ma: million years ago.

Node	Clade	Combined three-fossil and tectonics Scheme 1		Three-fossil Scheme 2		Four-fossil Scheme 3	
		Birth–death (Ma)	Yule (Ma)	Birth–death (Ma)	Yule (Ma)	Birth–death (Ma)	Yule (Ma)
A	Cycas crown	42.88 [57.76–33.45]	69.31 [116.82–34.53]	9.33 [15.65–3.11]	87.39 [149.81–32.99]	52.62 [63.46–47.50]	93.70 [155.47–47.52]
W	I	33.69 [36.30–32.00]	34.59 [36.97–32.23]	6.21 [10.00–1.66]	54.43 [97.56–18.82]	16.86 [26.37–8.63]	56.58 [101.44–22.47]
B	II–IV	27.14 [39.62–14.37]	51.38 [84.61–24.54]	6.82 [10.84–2.00]	64.83 [111.91–23.04]	23.83 [40.28–10.92]	69.89 [122.89–29.39]
C	II	17.97 [28.73–8.93]	36.47 [62.05–14.93]	4.94 [8.10–1.53]	45.58 [80.68–14.16]	14.71 [24.37–7.04]	48.07 [89.26–16.64]
D	III–IV	14.14 [20.95–8.09]	32.96 [54.32–14.72]	3.82 [5.30–1.16]	42.92 [75.18–12.00]	11.04 [16.64–6.01]	46.02 [85.56–17.19]

**Fig. 4.**
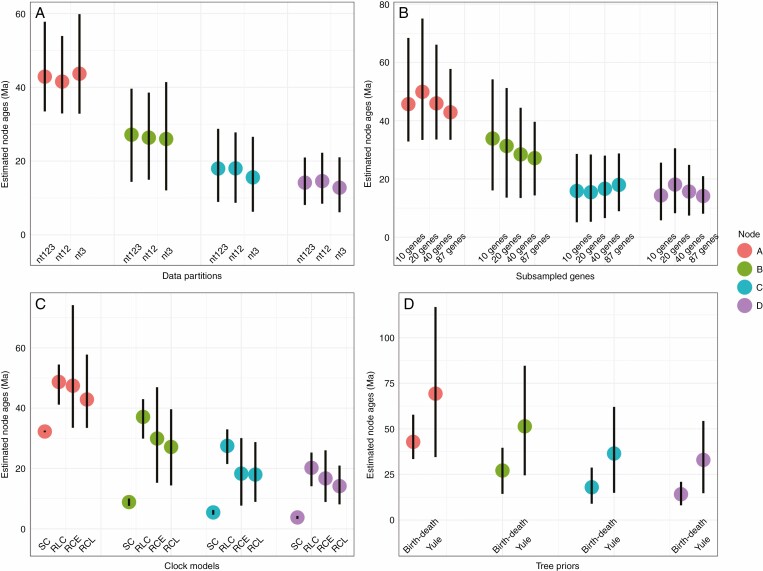
Comparisons of the ages inferred for important *Cycas* nodes across different analyses, based on different (A) dataset partitions, (B) subsamples of genes, (C) clock models and (D) tree priors. All analyses use calibration Scheme 1, uniform calibration priors, birth–death tree prior, uncorrelated relaxed clock lognormal model and all protein-coding genes unless otherwise stated. Abbreviations: nt123, a dataset including all nucleotide codons; nt12, dataset including the first and second positions of nucleotide codons; nt3, dataset only including the third position of nucleotide codons; the subsampled (10, 20, 40) genes from the PC dataset are listed in Supplementary Data Table S2; SC, strict clock; RLC, random local clock; RCE, uncorrelated relaxed clock exponential; RCL, uncorrelated relaxed clock lognormal. Refer to Fig. 3 for the major nodes A–D.

The PhyloMad analyses suggested a low risk in using the GTR+G+I substitution model for the datasets and indicated the third coding sites of the PC dataset had not reached saturation (Supplementary Data Fig. S8). This was consistent with the age estimation results by different data partitioning schemes, which showed little differences among different coding sites (Fig. 4A; Fig. S9). We found the estimated ages were also insensitive to the number of subsampled genes (Fig. 4B; Fig. S10). However, different clock models can lead to inconsistent results in age estimation: SC tended to have the youngest estimates; RLC exhibited the oldest mean estimates; and the two relaxed clock models showed similar estimates with RCL showing narrower confidential intervals than RCE (Fig. 4C; Fig. S11). Model test results supported the RCL model as the best-fit (Table 1). For the tree prior effect, the Yule prior led to significantly older ages than the birth–death prior for all major nodes (Fig. 4D; see also Table 2 for comparisons within different schemes), and model selection results favoured the birth–death prior for all schemes (Table S3).

### Biogeographical reconstruction

**Fig. 5.**
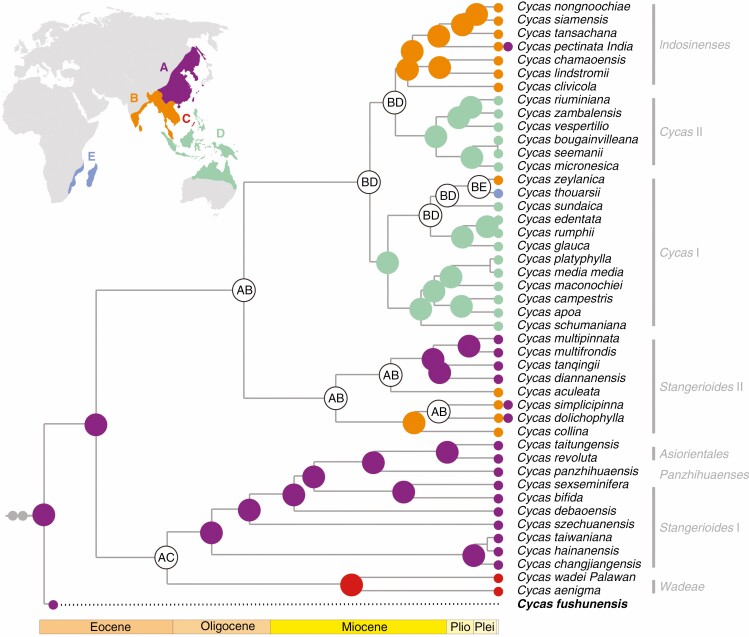
Biogeographical reconstruction using the statistical–dispersal–extinction–cladogenesis (S-DEC) method based on extant and fossil distributions of *Cycas*. The *Cycas fushunensis* clade is inserted as sister to the extant group, but set to being extinct after 1 million years for all 1000 randomly sampled posterior trees from BEAST. The tree is then summarized in RASP. Coloured dots at nodes represent most likely ancestral areas inferred by RASP. Inset is the geographical distribution of extant Cycadaceae (*Cycas*), including the five geographical regions used: A, East Asia, south to the Red River region; B, Indochina and India; C, Palawan and Culion islands; D, Southeast Asia islands including Australia; E, Africa. The two grey solid circles on the branch denote two extinct *Cycas* lineages (*C. cretacea* and *C. fujiiana*).

The DEC analyses with or without fossil integration produced incongruent results for crown Cycadaceae. The most probable ancestral area of Cycadaceae was inferred as East Asia (region A) when the fossil distribution was taken into consideration (Fig. 5), while the crown node remained ambiguous (regions AB) when only extant *Cycas* was used for inference (Supplementary Data Fig. S12).

## Discussion

### Impacts of different effects on age estimation

A previous study of angiosperms suggested the evolutionary timescale was robust to large reductions in the number of genes, and to substantial changes of most models and priors (Foster *et al.*, 2017). Here, the *Cycas* timescale was generally insensitive to the number of sampled genes. We also found that our estimate of the *Cycas* evolutionary timescale was robust to the partitioned codons. This was not surprising, as the protein-coding sequences used in this study have not reached saturation to impact the results. However, age estimates of Cycadaceae remain dependent on the choice of clock models, with SC generally leading to younger node ages than the other clock models, and RLC yielding slightly older ages. The strict clock can perform well for trees with shallow roots because of low levels of rate variation between branches (Brown and Yang, 2011), but this is not the case in analyses of most empirical datasets (Crisp *et al.*, 2014) nor in this study. Because of use of a constant rate, the 95% credibility interval based on the SC model was found to be much narrower, as expected based on previous studies (Ho *et al.*, 2005; Foster *et al.*, 2017). The RLC also yielded more precise confidential intervals but slightly older ages than the relaxed clocks. Previous studies suggested that RLC would perform better for datasets with clade-specific substitution rates (e.g. Crisp *et al.*, 2014). However, there seemed to be no significant fast or slowly evolving clades for our ingroup dataset (Supplementary Data Fig. S2). Hence, the ages inferred from the two clock models (SC and RLC) were unreliable as these models were rejected (Table 1). Both of the relaxed clock models (RCL and RCE) led to similar ages for crown *Cycas* (~43 vs. 47 Ma, respectively), and the RCL showed more precise estimations and was favoured by the model selection. These potential inconsistent results indicate that the suitability of clock models should be compared by using a priori criteria (see Crisp *et al.*, 2014) or a posteriori marginal likelihood test.

Previous studies have also documented that the branching process prior can substantially impact the age estimation for lineages that have undergone massive extinction [e.g. in cycads (Condamine *et al.*, 2015), *Xanthorrhoea* (Crisp *et al.*, 2014) and *Ephedra* (Rydin *et al.*, 2021)], suggesting the tree prior effects are strongly dependent on data characteristics and the underlying phylogeny (Ritchie *et al.*, 2017). The Yule prior would, in general, be expected to yield an older age for the crown age compared to the birth–death prior because the Yule prior assumes a constant birth rate and zero extinction, resulting in nodes being more evenly spread over the tree (Rydin *et al.*, 2021). Hence, the birth–death prior estimates more nodes toward the present compared to the past (Gernhard, 2008), leading to a ‘pull to the present’ effect. Given this, we expected contrasting results for all nodes in different schemes (see Table 2, Fig. 2 and Supplementary Data Fig. S4) during age estimation for extant *Cycas* based on different tree priors (i.e. Yule vs. birth–death). The long extinction history of *Cycas* may be more sensitive to branching process priors than the extinction history of other plant groups (Nagalingum *et al.*, 2011; Condamine *et al.*, 2015).

We also found that the estimated node ages of crown Cycadaceae based on the same birth–death prior and similar fossils were younger in this study (<10 Ma, Scheme 2, Table 2; Supplementary Data Fig. S5a) than all previous studies (Nagalingum *et al.*, 2011; Salas-Leiva *et al.*, 2013; Condamine *et al.*, 2015; Mankga *et al.*, 2020) (all >10 Ma). This may be caused by the nuclear markers used in previous molecular dating studies evolving faster than the plastid genes employed in the present study. Despite some differences, these results all suggested a Late Miocene diversification of Cycadaceae, which was significantly younger than the estimated ages by the Yule prior in different schemes (Fig. 2). This was especially significant for Scheme 2 of this study where only deep nodes were constrained, yielding the age of crown *Cycas* varying from ~10 to 87 Ma (Table 2). This variation in age estimation between different priors revealed an eight-fold difference, which was greater than the three-fold difference in general revealed in a previous comparison (Condamine *et al.*, 2015). Considering the different sampling fractions, sequence and fossil matrices used between previous studies and our study, it is difficult to offer a reasonable explanation for why there were wider differences between Yule and birth–death priors in the present study. However, these results may suggest that the influence of tree priors on molecular dating may be greater than previously thought.

A birth–death model with homogeneous rates across time and various clades within large groups is still an oversimplified model (Condamine *et al.*, 2015), and the extinction rates inferred by molecular phylogeny based only on extant lineages should also be interpreted cautiously (Marshall, 2017). In this study, we found the variation between different branching process priors can be mitigated by introducing internal calibrations (Fig. 2; Supplementary Data Fig. S4: Scheme 2 vs. 1 and 3), which may be explained by the interruption of constant extinction processes from the stem to tips. This explanation was supported by the results of our extinction rates estimation, which revealed greater than two-fold extinction rates in Scheme 2 than the others. We also found the cross-validation can yield inconsistent results under the same branching process prior. Specifically, using the birth–death prior, integrating the biogeographical calibration (37–32 Ma, Scheme 1) yielded a close mean crown age to the fossil (43 vs. 47.5 Ma). However, constraining the crown node using a fossil calibration as a minimum age (47.5 Ma, Scheme 3) yielded a younger estimate for the biogeographically calibrated node than expected (16.6 vs. 37–32 Ma, Table 1), indicating the performance of different calibration schemes will not always be similar by cross-validation (Warnock *et al.*, 2015). Given the above concerns, we recommend careful selection of both priors and fossils (or examined biogeographical events, if available) for internal calibration before inferring timescales of lineages that have undergone massive extinctions. Indeed, these cases indicate lineage age estimation will probably yield substantial gaps by using different priors or fossil combinations in BEAST (Rydin *et al.*, 2021).

### *Crown ages of* Cycas *under different schemes*

Recent empirical studies by molecular dating have reaffirmed that many lineages (Cook and Crisp, 2005; Giles *et al.*, 2017) or floras (Chen *et al.*, 2018) may be younger than previously thought. Yet because of an erroneous ‘prior belief’ or impositions from results (Heads, 2012), clade ages inferred or interpreted from the molecular clock are often too young to have been affected by the tectonic events that coincide spatially with the clades’ distributions. This apparent discrepancy is used to discount vicariance and support a dispersal model (Heads, 2019). While any empirical study must consider the fossil records as a priority when estimating the timescale of the tree of life (and this will provide minimum ages), one should also take the phylogenetic clades and their distribution into consideration, as these are the strongest components of molecular phylogenetics (Heads, 2019). The previous phylogenetic framework of Cycadaceae revealed an ambiguous position of section *Wadeae*, possibly caused by the limited and incongruent molecular markers employed (Liu *et al.*, 2018). However, our phylogeny inferred by plastid genomic data explicitly resolved the *Wadeae* group as sister to the Southeast Eurasian clade comprising sections *Panzhihuaenses*, *Asiorientales* and *Stangerioides* (Fig. 3). This result was consistent with the morphological resemblance among these sections (Hill, 1999) as well as the phylogeny based on nrITS orthologues (Xiao and Möller, 2015), which further conformed to the previously proposed vicariance-for-speciation hypothesis for this section (Tang, 2004). In this study, we offered different calibration schemes, aiming to compare and discuss the evolutionary histories (dispersal or vicariance) of the *Wadeae* group under different timelines.

The scenario that extant *Cycas* diversified after the Late Miocene, as indicated by all previous molecular estimates using a birth–death tree prior (e.g. Nagalingum *et al.*, 2011; Condamine *et al.*, 2015; Scheme 2 of this study), leaves us with a very recent divergence of the *Wadeae* group (6.21 Ma, 95% HPD: 10.00–1.66 Ma). Despite representing a minimum age, this timeline can largely exclude the possibility of vicariance, as the rifting of Palawan (Hall, 2002) is much older than the upper boundary of the 95% credible interval of the divergence time (10 Ma, Table 2). If the current distribution of the *Wadeae* group in the Palawan islands is a result of recent dispersal from South China rather than vicariance events, the group must have dispersed very quickly over large parts of the South China Sea via two possible routes. First is the South China–Southwest China–Indochina–Borneo route when Palawan and other micro-continents had collided with Borneo ~10–5 Ma (Karig *et al.*, 1986) in its current position (Hall, 2009). However, *Cycas* taxa normally have large and heavy seeds with limited dispersal ability (Dehgan and Yuen, 1983; Xiao and Möller, 2015; but see the coastal *Rumphiae* group: Keppel *et al.*, 2008; Liu *et al.*, 2021), and thus the *Wadeae* group lacks the dispersal mechanism to migrate over such long distances within such a short time (6.21 Ma on average; Table 2; Supplementary Data Fig. S5a). The second route is a migration shortcut scenario from Taiwan via Luzon to Palawan (Esselstyn and Oliveros, 2010), although this was also unlikely due to the deep sea arc between Taiwan and Luzon islands (>1000 m, Heaney, 1985), and no emergent island has ever been present in the South China Sea (Hall, 2009). Moreover, the fact that no sister lineages of the *Wadeae* group were found anywhere in Indochina, Borneo and Luzon also supports the rejection of these routes.

On the other hand, a previous study suggested that crustal deformation, and environmental and climatic changes related to orogenic events probably stimulated the lineage separation and speciation of Cycadaceae through vicariance events (Xiao and Möller, 2015). The vicariance hypothesis can be favoured in explaining the occurrence of section *Wadeae* in Palawan, given the limited dispersal ability of this group and its strong plastid phylogenetic affinity with continental Eurasia (Fig. 3). This tectonic rifting event, which contributed to the previously proposed ‘Palawan Arc’ hypothesis (Blackburn *et al.*, 2010; Siler *et al.*, 2012), considers that Eurasian micro-continental blocks on the edge of the Eurasian plate carried floral and faunal assemblages when they split and rafted from the Asian main landmass and collided with the Philippine mobile belt after the Late Eocene to Early Oligocene (Fig. 1). To our knowledge, we offer the first case to comprehensively demonstrate the ‘Palawan Arc’ hypothesis in plants. The date derived by combining this vicariance event in molecular dating pre-dates the crown node of Cycadaceae from the Late Miocene (Nagalingum *et al.*, 2011; Condamine *et al.*, 2015) to the Eocene (Fig. 3, Table 2). This is further congruent with the stratigraphic ages of multiple fossils assigned to this genus characterized by the obvious single vein midrib (Yokoyama, 1911; Krassilov, 1978; Su *et al.*, 2014).

Previous molecular dating studies supporting post-Miocene diversification of extant cycads have questioned the validity of the Palaeogene putative *Cycas* fossils, due to the extensive infrageneric homoplasy found in the leaflet and cuticle characters within extant *Cycas* (Griffith *et al.*, 2014). They argued because of this anatomical homoplasy, these cuticle characters cannot be used to link the fossils to the extant genus (Condamine *et al.*, 2015). However, no details were given to justify their argument. On the contrary, the leaflet anatomical characters have recently been shown to reveal a substantial phylogenetic signal in extant cycad evolution at the supra-generic level (Coiro *et al.*, 2020). Moreover, the unique single-vein midrib character (with one vascular bundle) displayed in those ‘questionable’ cycad fossils (Yokoyama, 1911; Krassilov, 1978; Su *et al.*, 2014), as a gross morphological feature of leaflets, is believed to have evolved independently (Hermsen *et al.*, 2007; Griffith *et al.*, 2014) and has not been found in any cycad fossil or extant group except *Cycas* (Hermsen *et al.*, 2006). Thus, the anatomical traits can be valid for generic-level identification, and it should be reasonable to assign these Palaeocene cycad fossils (Yokoyama, 1911; Krassilov, 1978; Su *et al.*, 2014) to the genus *Cycas*, at least based on their shared exclusive midrib trait from a botanical and palaeobotanical perspective.

Dating methods limited to only a few fossils are highly sensitive to the assignment of calibrations and defined priors, where assignments can lead to biased substitution rate estimates (Duchêne *et al.*, 2014), and poor fossil calibration coverage of certain lineages when more fossil calibrations are available may lead to underestimated node ages (Sauquet *et al.*, 2012; Wang and Mao, 2016). This implies the four- or six-fossil datasets previously used for deep node calibrations to rebuild the timescale of extant cycads (Nagalingum *et al.*, 2011; Condamine *et al.*, 2015) remain insufficient. A growing number of convincing cycad fossils could be used for internal calibration by assigning to extant genera, such as *Cycas* from the Eocene (Su *et al.*, 2014), *Zamia* from the Eocene–Oligocene (Erdei *et al.*, 2018), *Ceratozamia* from the Oligocene (Kvaček, 2014); or very close to an extant group (e.g. *Eobowenia* from the Early Cretaceous, Coiro and Pott, 2017). Therefore, the radiation of cycad clades established by previous molecular studies (Nagalingum *et al.*, 2011; Condamine *et al.*, 2015) could have been underestimated not only because of a lack of fossil or biogeographical support, but also because they were influenced by the tree priors owing to the absence of effective internal calibrations (discussed above).

Apart from the evidence from our molecular dating based on biogeographical calibration, the post-Miocene diversification of cycads is also questioned by evidence from the long beetle–cycad evolutionary history that can be dated back to the Early Jurassic (Cai *et al.*, 2018). A recent molecular dating study on another cycad genus, *Dioon*, used biogeographical calibrations and also yielded a Palaeogene age of 56 Ma for the crown node (Gutiérrez-Ortega *et al*., 2018), which is significantly older than that without considering tectonic events (~14 Ma) (Condamine *et al.*, 2015). Together, these studies challenge the view that diversification of extant cycad genera took place after the Neogene (Nagalingum *et al.*, 2011; Condamine *et al.*, 2015).

### *Biogeographical implications for* Cycas

The ancestral area of Cycadaceae was recently inferred as a tropical region (i.e. Indochina) (Mankga *et al.*, 2020), overturning the conclusion from a previous study that suggested subtropical South China (Xiao and Möller, 2015). Using our plastomic phylogeny, we found the inferred ancestral area for *Cycas* based on the extant group is ambiguous (Supplementary Data Fig. S12), while including the fossil distribution can extend the ancestral area to a wide but explicit East Asia (Fig. 5), rejecting the Indochina origin hypothesis. The reason why the previous study proposed Indochina as the ancestral area is because of the non-monophyletic section *Indosinenses* revealed in their phylogeny (Mankga *et al.*, 2020). However, their result is not supported by morphology or geography, and is incongruent with the present and all other previous phylogenies (Hill, 1999; Nagalingum *et al.*, 2011; Condamine *et al.*, 2015; Xiao and Möller, 2015), even the one from which they obtained the source data (Liu *et al.*, 2018). Thus, their conclusion on this basis is unconvincing.

The monophyly of section *Stangerioides* was supported by morphology (Hill, 2008) and proven by the nrITS phylogeny (Xiao and Möller, 2015). However, neither the previous phylogeny (Liu *et al.*, 2018) involving chloroplast markers nor the plastid phylogenomic tree in the present study supports the monophyly of section *Stangerioides* (Fig. 3; Supplementary Data Fig. S2), from which the two subclades of this section roughly diverged at the boundary of the Red River Fault, which is recognized as one of the main geological discontinuities of Southeast Asia (Leloup *et al.*, 1995). The clear biogeographical pattern of *Stangerioides* revealed in this study suggests a plastid divergence triggered by this geographical barrier that formed during Oligocene to Miocene (Tapponnier *et al.*, 1982; Leloup *et al.*, 1995). More broadly, this geographical isolation has also promoted the plastid divergence within *Cycas* between Clade I and Clades II–IV (Fig. 3), as no lineage from Clade I has crossed the barrier.

Our phylogeny also suggests the close relationship between sections *Cycas* and *Indosinenses*, which revealed a sister clade of *Indosinenses* and some lineages from sections *Cycas* in the Pacific (Fig. 3). A similar close affinity was found between the Australian species and some lineages from the Indian Ocean. Previous studies have suggested that long-distance dispersal of *Cycas* could have shaped the current distribution of the genus (Keppel *et al.*, 2008; Xiao and Möller, 2015; Liu *et al.*, 2021). The lineages from many remote Pacific islands (Guam, New Britain and Vanuatu, Fig. 3) were revealed as sisters to section *Indosinenses* from the Indochina Peninsula, suggesting that seed dispersal via ocean currents may contribute to the gene flow over these regions (Keppel *et al.*, 2008; Liu *et al.*, 2021). However, more comprehensive sampling is required to unravel the specific mechanism in driving these patterns in *Cycas*.

## Conclusions

Extant Cycadaceae is believed to have undergone recent and post-Neogene radiation in the context of long historical mass extinctions. However, our updated phylogeny based on plastomic data challenges this view by combining extra evidence from plate tectonics and the geological age of fossils assigned to *Cycas*. Molecular dating by integrating biogeographical calibration robustly indicates that crown *Cycas* dates back to the Palaeogene, and strongly supports the idea that previously described *Cycas* fossils represent extinct sisters of the extant group. Biogeographical analysis incorporating fossils rejects the Indochina origin of *Cycas* and instead assigns the ancestral area of this group to a more extensive East Asia region. This study highlights the importance of combining phylogenetic clades, tectonic events and fossil records for inferring the evolutionary history of lineages that have undergone massive extinctions.

## SUPPLEMENTARY DATA

Supplementary data are available online at https://academic.oup.com/aob and consist of the following. Fig. S1: Tanglegrams of maximum-likelihood trees of *Cycas* using whole plastomic data and protein-coding genes in this study. Fig. S2: Phylogram of the maximum-likelihood tree of *Cycas* based on the whole chloroplast genomic dataset using IQTREE in this study. Fig. S3: Comparison between the maximum-likelihood tree based on the whole chloroplast genomic dataset and BEAST maximum clade credibility tree based on the protein-coding region dataset of *Cycas* in this study. Fig. S4: Density plots of marginal posterior age distributions of the *Cycas* crown node based on three calibration schemes and different tree priors. Fig. S5: Chronogram depicting the *Cycas* evolutionary timescale, as estimated by birth–death and Yule priors under calibration Scheme 2 based on 87 protein-coding genes using Bayesian analysis with an uncorrelated relaxed clock, in BEAST. Fig. S6: Chronogram depicting the *Cycas* evolutionary timescale, as estimated by the Yule prior under calibration Scheme 1 based on 87 protein-coding genes using Bayesian analysis with an uncorrelated relaxed clock, in BEAST. Fig. S7: Chronogram depicting the *Cycas* evolutionary timescale, as estimated by birth–death and Yule priors under calibration Scheme 3 based on 87 protein-coding genes using Bayesian analysis with an uncorrelated relaxed clock, in BEAST. Fig. S8: Corrected vs. uncorrected pairwise differences for first and second codon positions, and third codon positions for the 87-gene dataset. Fig. S9: Chronogram depicting the *Cycas* evolutionary timescale, as estimated by the birth–death prior under calibration Scheme 1 based on the partitioned datasets with the first and second positions of the nucleotide codon, and only the third positions of the nucleotide codon, using Bayesian analysis with an uncorrelated relaxed clock, in BEAST. Fig. S10: Chronogram depicting the *Cycas* evolutionary timescale, as estimated by the birth–death prior under calibration Scheme 1 based on a subsample of 10, 20 and 40 protein-coding genes using Bayesian analysis with an uncorrelated relaxed clock, in BEAST. Fig. S11: Chronogram depicting the *Cycas* evolutionary timescale, as estimated by the birth–death prior under calibration Scheme 1 based on 87 protein-coding genes using strict clock, random local clock and uncorrelated relaxed clock exponential models by Bayesian analysis in BEAST. Fig. S12: Biogeographical reconstruction based on the statistical–dispersal–extinction–cladogenesis method and distributions of extant *Cycas*. Table S1: Collection information, vouchers, plastomic characteristics and NCBI accessions of the *Cycas* samples used in this study. Table S2: Information of randomly subsampled protein-coding genes in each scheme used for BEAST analyses. Table S3: Marginal likelihood estimates of Yule and birth–death priors for different calibration scenarios inferred by the nested sampling method in BEAST. Table S4: Area adjacent matrix used in ancestral reconstruction analyses.

mcab118_suppl_Supplementary_Materials_S1

mcab118_suppl_Supplementary_Materials_S2
