# Supplementary material for: Not that young: combining plastid phylogenomic, plate tectonic and fossil evidence indicates a Palaeogene diversification of Cycadaceae
Source: Ann Bot. 2021 Sep 14;129(2):217–30. doi: 10.1093/aob/mcab118 (PMC8796677; doi:10.1093/aob/mcab118)
Supplement: mcab118_suppl_Supplementary_Materials_S1 [file mcab118_suppl_supplementary_materials_s1.docx]

**SUPPLEMENTARY TABLES**

**Table S1** Collection information, vouchers, plastomic characteristic and NCBI accessions of the *Cycas* samples used in this study. NA: Sampled plastomes from NCBI with collection information not available.

| Species | Locality | Voucher | Herbarium deposited | Total length of the plastome (bp) | NCBI accession |
| --- | --- | --- | --- | --- | --- |
| *Cycas aculeata* | Vietnam | AC26 | KUN | 162003 | MZ339196 |
| *Cycas aenigma* | Palawan, Philippines | 82 | BK | 162140 | MZ339189 |
| *Cycas apoa* | Paup New Guinea | 47 | BK | 162056 | MZ339182 |
| *Cycas bifida* | Guangxi, China | hhd01 | KUN | 162128 | MZ339195 |
| *Cycas bougainvilleana* | New Britain, Paup New Guinea | 11256 | BK | 162098 | MW713704 |
| *Cycas campestris* | Australia | 60M | BK | 162037 | MZ339193 |
| *Cycas chamaoensis* | Thailand | 63B | BK | 162000 | MZ339184 |
| *Cycas changjiangensis* | Hainan, China | 417 | BK | 162224 | MZ339176 |
| *Cycas clivicola* | Malaysia | 11279 | BK, W | 161999 | MZ339183 |
| *Cycas collina* | Vietnam | CPC8251 | KUN | 161854 | MZ339179 |
| *Cycas debaoensis* | NA | NA | NA | 162092 | KU743927 |
| *Cycas debaoensis* | NA | NA | NA | 162094 | KM459003 |
| *Cycas diannanensis* | Yunnan, China | mhg12 | KUN | 162013 | MZ339165 |
| *Cycas dolichophylla* | Vietnam | 163 | BK | 161838 | MZ339170 |
| *Cycas edentata* | Cebu, Philippines | 19373 | BK | 162099 | MW713694 |
| *Cycas glauca* | Timor, Indonesia | 10621 | BK | 162099 | MZ339181 |
| *Cycas hainanensis* | Hainan, China | 91 | BK | 162215 | MZ339171 |
| *Cycas lindstromii* | Vietnam | 96 | L | 162061 | MZ339185 |
| *Cycas maconochiei* ssp *viridis* | Australia | 65 | BK | 162164 | MZ339166 |
| *Cycas media media* | Australia | 461 | L, BK | 162106 | MZ339192 |
| *Cycas micronesica* | Guam | 19295 | BK | 162086 | MW713699 |
| *Cycas multifrondis* | China | FZMF1 | KUN | 161993 | MZ339191 |
| *Cycas multipinnata* | Yunnan, China | hhdmp1 | KUN | 162060 | MZ339174 |
| *Cycas nongnoochiae* | Thailand | TNN1 | BK | 162096 | MZ339169 |
| *Cycas panzhihuaensis* | NA | NA | NA | 162470 | KX713899 |
| *Cycas pectinata* | India | 123A | L | 162086 | MZ339190 |
| *Cycas platyphylla* | Australia | 466 | L, BK | 162051 | MZ339178 |
| *Cycas revoluta* | NA | NA | NA | 162489 | JN867588 |
| *Cycas riuminiana* | Philippines | 11141 | L | 162101 | MZ339167 |
| *Cycas rumphii* | Sulawesi, Indonesia | 11156 | L | 162098 | MW713712 |
| *Cycas schumaniana* | Paup New Guinea | 11157 | L | 162002 | MZ339186 |
| *Cycas seemanii* | Tanna, Vanuatu | 11278 | BK | 162093 | MW713728 |
| *Cycas sexseminifera* | Guangxi, China | 113 | BK | 162107 | MZ339172 |
| *Cycas siamensis* | Thailand | SA1 | BK | 162117 | MZ339177 |
| *Cycas simplicipinna* | Choi, Thailand | NNSI1 | Cultivated sample | 161961 | MZ339180 |
| *Cycas sundaica* | Flores, Indonesia | 10627D | BK | 162099 | MW713727 |
| *Cycas szechuanensis* | NA | NA | NA | 162083 | NC042668 |
| *Cycas taitungensis* | NA | NA | NA | 163403 | AP009339 |
| *Cycas taitungensis* | NA | NA | NA | 163403 | NC009618 |
| *Cycas taiwaniana* | Hainan, China | 98A | BK | 162204 | MZ339194 |
| *Cycas tanqingii* | Yunnan, China | 480A | BK | 162060 | MZ339173 |
| *Cycas tansachana* | Thailand | 157B | BK | 162058 | MZ339187 |
| *Cycas thouarsii* | Madagascar | 148E | BK | 161753 | MW713696 |
| *Cycas vespertilio* | Negros, Philippines | 19441 | BK | 162105 | MZ339188 |
| *Cycas wadei* | Culion, Philippines | 150A | BK | 161632 | MZ339175 |
| *Cycas zambalensis* | Lvzon, Philippines | NNZM1 | Cultivated sample | 162085 | MZ339168 |
| *Cycas zeylanica* | Andaman, India | 11270 | BK | 161756 | MW713697 |

**Table S2** Information of randomly subsampled protein-coding genes in each scheme (10, 20, and 40 gene datasets) used for BEAST analyses.

| Different gene number sets | Randomly selected genes |
| --- | --- |
| 10 | *accD, atpH, ndhD, ndhJ, psbB, psbK, rpl16, rpoC1, rps4, ycf3* |
| 20 | *atpA, cemA, clpP, ndhD, ndhI, ndhK, petA, petB, psaA, psbA, psbB,*  *psbI, rbcL, rpl16, rpl20, rpoC1, rps3, rps4, rps19, ycf4* |
| 40 | *accD atpA, atpF, atpH, cemA, clpP, ndhC, ndhD, ndhG, ndhH, ndhI, ndhJ, ndhK, petA, petB, petL, psaA, psaB, psbA, psbB, psbC, psbH, psbI, psbK, psbL, rbcL, rpl2, rpl16, rpl20, rpl22, rpoA, rpoC1, rps14, rps19, rps2, rps3, rps4, rps8, ycf3, ycf4* |

**Table S3** Marginal likelihood estimates of Yule and birth-death priors for different calibration scenarios inferred by nested sampling method in BEAST. The favored prior is bolded if the likelihood is greater than the other by two folds of the standard deviation (SD).

|  | Combined three-fossil and biogeographic calibration  (Scheme 1) | | Three-fossil calibration  (Scheme 2) | | | Four-fossil calibration  (Scheme 3) |  |
| --- | --- | --- | --- | --- | --- | --- | --- |
| Tree priors | **Birth-death**  (SD =2.01) | Yule  (SD = 2.01) | | **Birth-death**  (SD = 2.00) | Yule  (SD = 2.01) | **Birth-death**  (SD = 2.01) | Yule  (SD = 2.01) |
| Marginal likelihood | -221605.14 | -222426.74 | | -222721.24 | -223720.62 | -223103.23 | -223944.66 |

**Table S4** Area adjacent matrix used in ancestral reconstruction analyses. The number ‘1’ represents that area pairs are adjacent and ‘0’ represents pairwise areas are not adjacent. A: East Asia; B: Indochina and India (including Sri Lanka); C: Palawan and Culion Islands; D: Pacific Islands and Australasia; and E: Africa.

|  | **A** | **B** | **C** | **D** | **E** |
| --- | --- | --- | --- | --- | --- |
| **A** | - | 1 | 1 | 0 | 0 |
| **B** | 1 | - | 1 | 1 | 1 |
| **C** | 1 | 1 | - | 1 | 0 |
| **D** | 0 | 1 | 1 | - | 0 |
| **E** | 0 | 1 | 0 | 0 | - |
