# Supplementary material for: Not that young: combining plastid phylogenomic, plate tectonic and fossil evidence indicates a Palaeogene diversification of Cycadaceae
Source: Ann Bot. 2021 Sep 14;129(2):217–30. doi: 10.1093/aob/mcab118 (PMC8796677; doi:10.1093/aob/mcab118)

**SUPPLEMENTARY FIGURES**

**Fig. S1.** Tanglegrams of Maximum likelihood (ML) trees of *Cycas* using A: whole plastomic (WP) data and B: protein-coding genes (PC) in this study. Grey lines linked the same taxa. Both the topologies were inferred by IQTREE. Colored dots on the nodes represent different ranges of bootstrap percentages (BP).


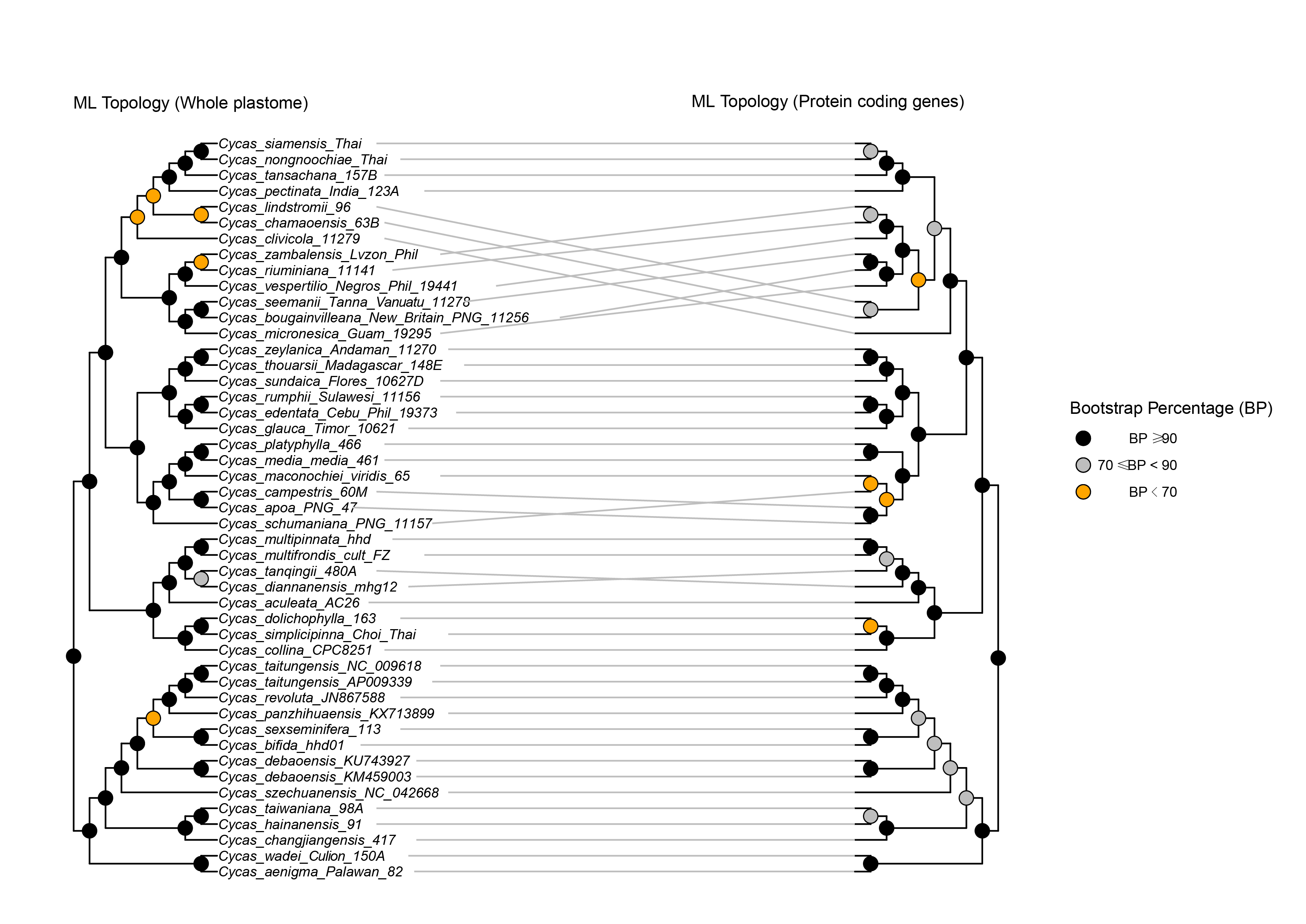


**Fig. S2.** Phylogram of Maximum likelihood (ML) tree of *Cycas* based on whole chloroplast genomic dataset using IQTREE in this study. Colored dots on the nodes represent different ranges of bootstrap percentages (BP).

**
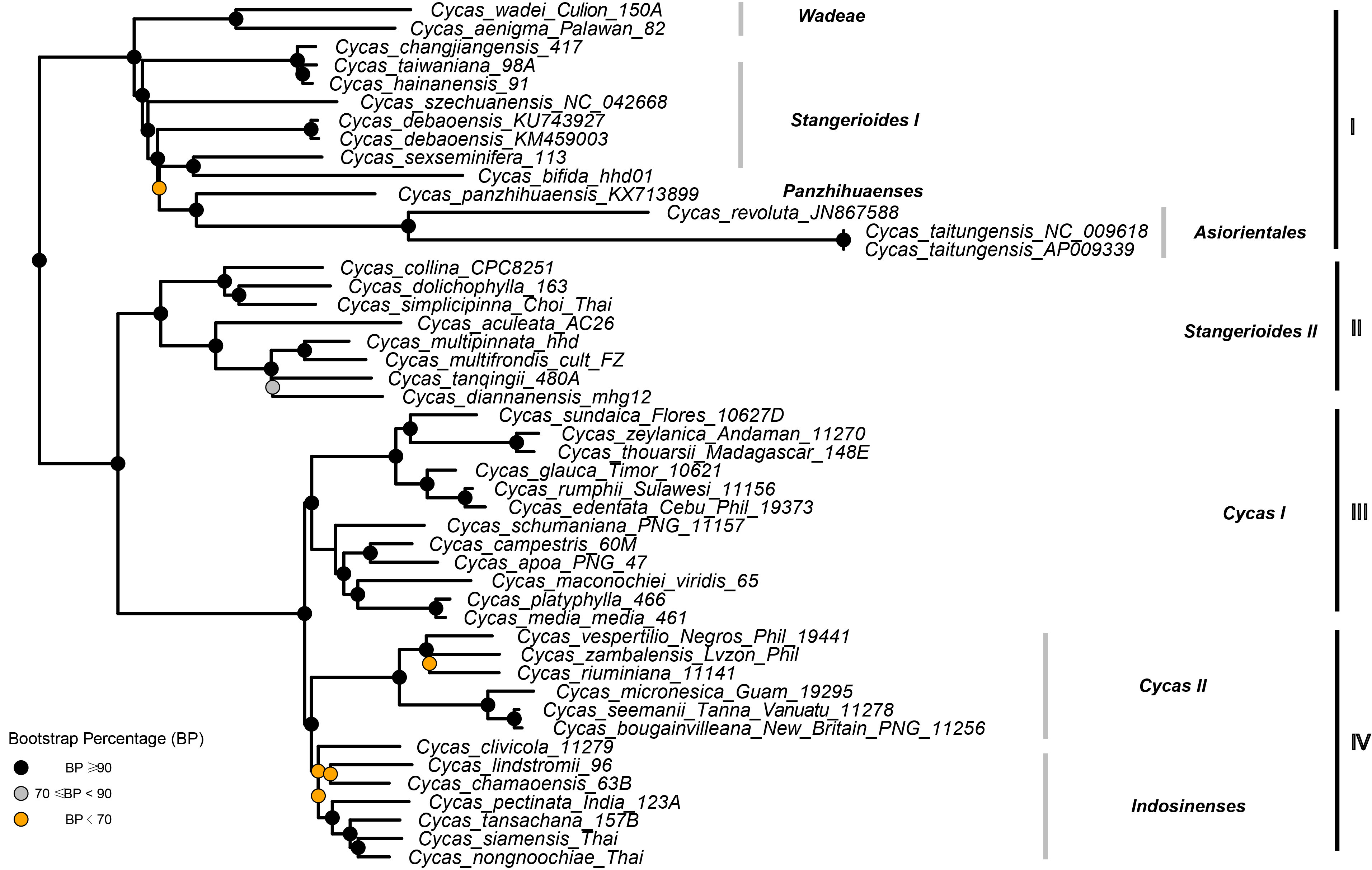
**

**Fig. S3.** Comparison etween Maximum likelihood (ML) tree based on whole chloroplast genomic dataset and BEAST maximum clade credibility (MCC) tree based on protein-coding region dataset of *Cycas* in this study. Grey lines linked the same taxa. Colored nodes represent different ranges of bootstrap percentages (BP) for the ML tree and posterior probabilities (PP) for the MCC tree.

**
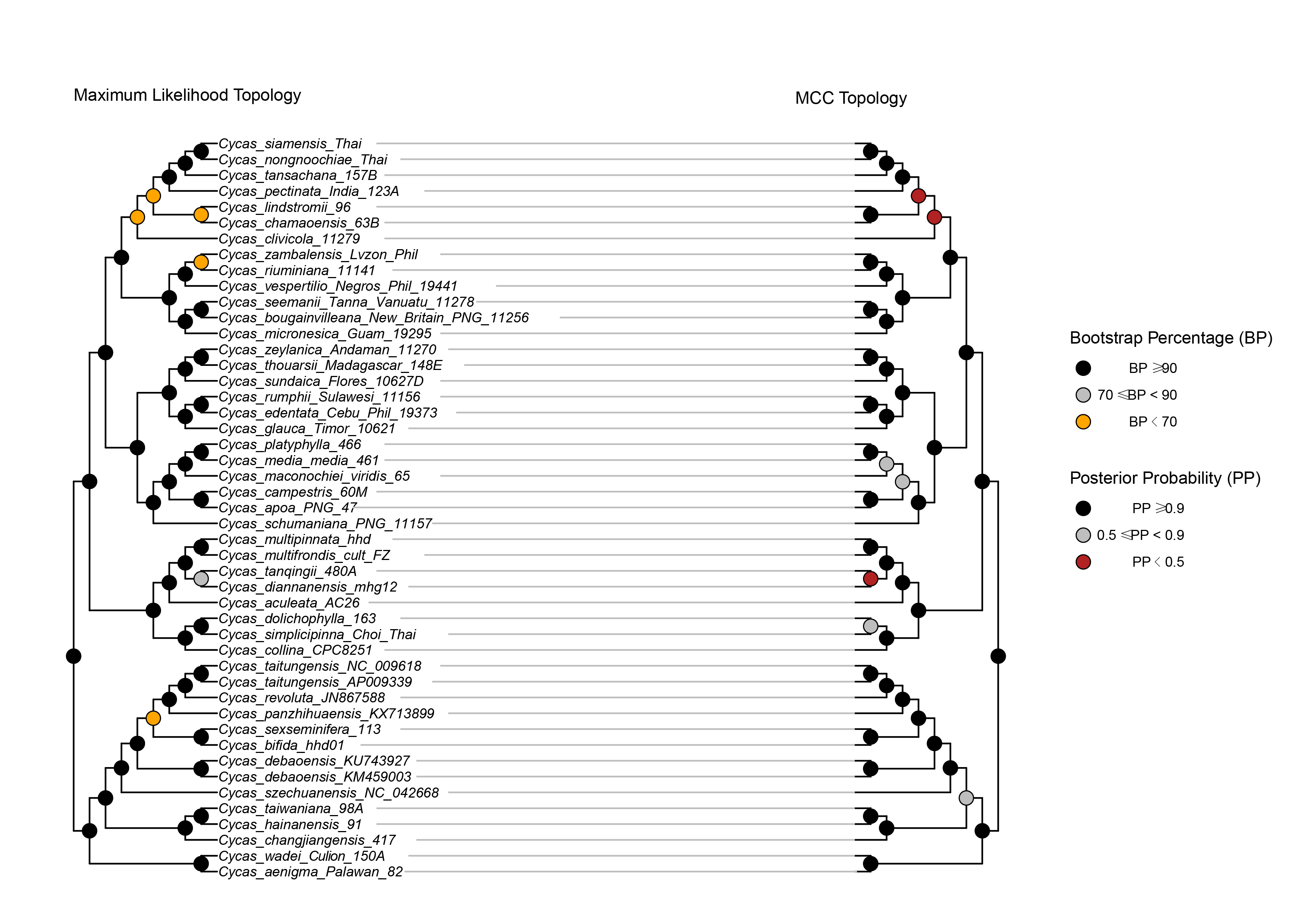
**

**Fig. S4.** Density plots of marginal posterior age distributions of *Cycas* crown node based on three calibration schemes (Schemes 1–3) and different tree priors (birth-death and Yule). Ages were extracted from 10,000 posterior trees from BEAST analyses. Refer to Figure 3 for the major nodes of B–D.

**
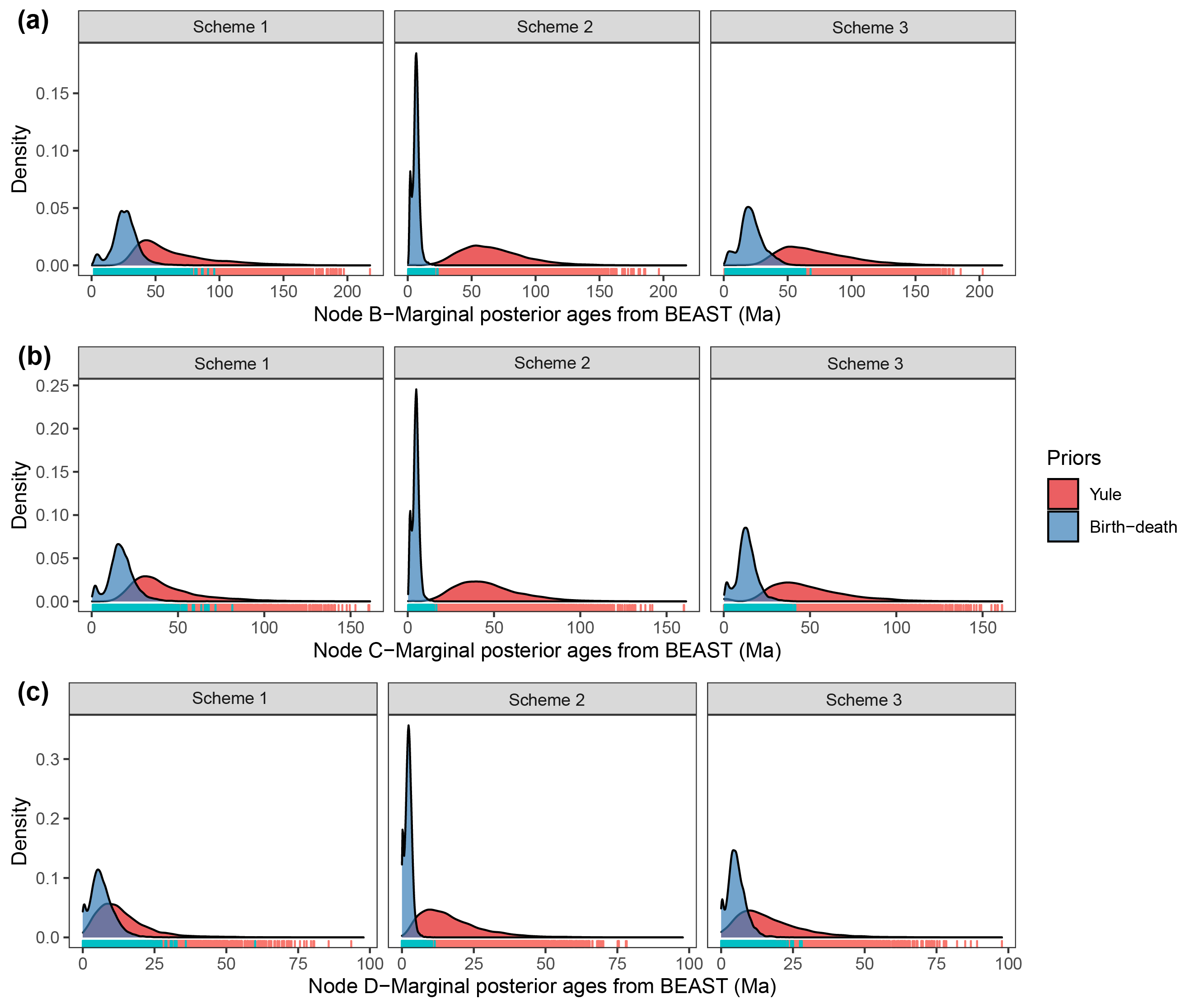
**

**Fig. S5.** Chronogram depicting the *Cycas* evolutionary timescale, as estimated by (a) birth-death and (b) Yule prior under calibration Scheme 2 based on 87 protein-coding genes using Bayesian analysis with an uncorrelated relaxed clock, in BEAST. Mean ages (in millions of years) are indicated for nodes, with grey bars indicating the 95% credibility intervals for these nodes. Morphologically recognized sections are indicated within the clades. Outgroups are grey shaded.

**
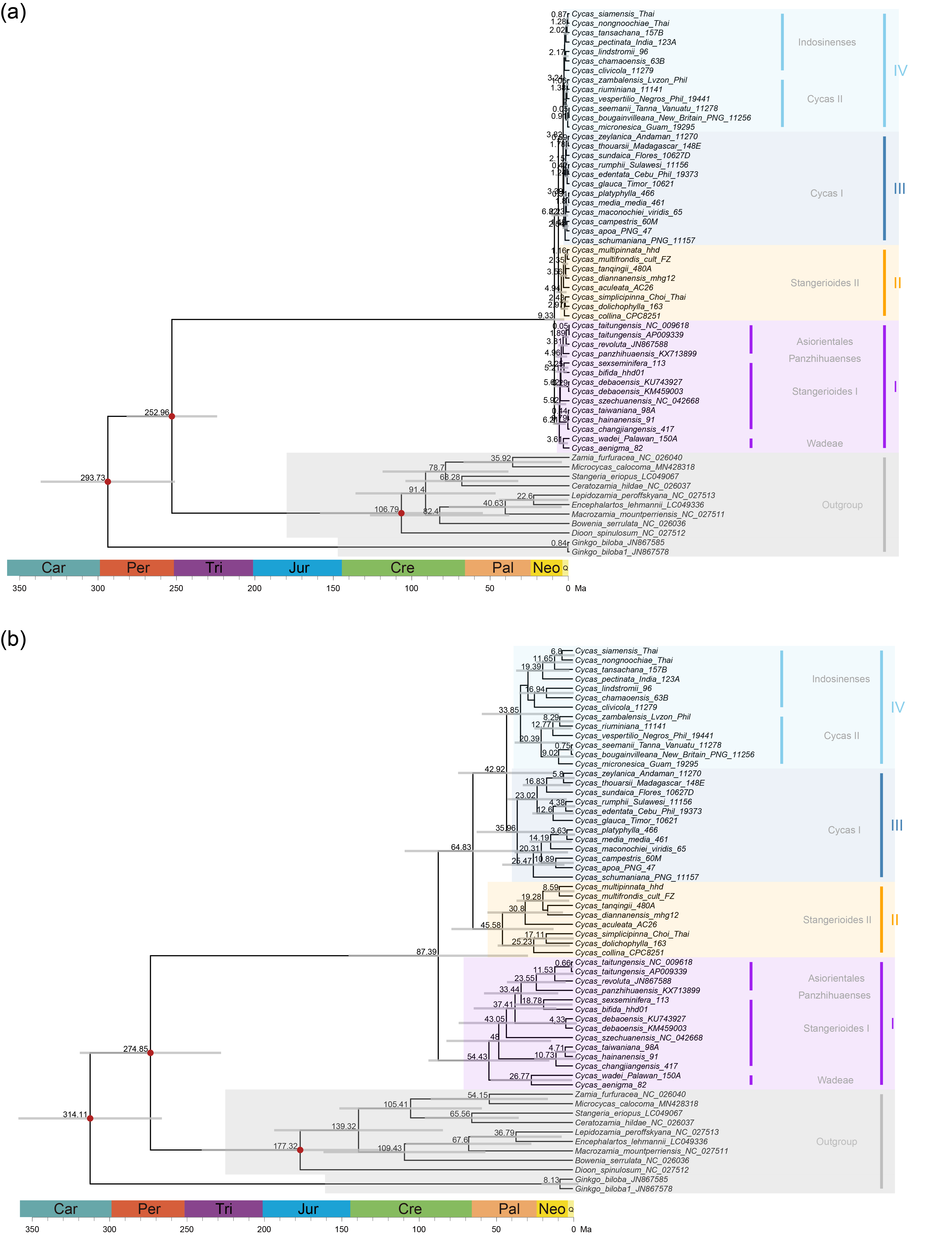
**

**Fig. S6.** Chronogram depicting the *Cycas* evolutionary timescale, as estimated by Yule prior under calibration Scheme 1 based on 87 protein-coding genes using Bayesian analysis with an uncorrelated relaxed clock, in BEAST. Mean ages (in millions of years) are indicated for nodes, with grey bars indicating the 95% credibility intervals for these nodes. Morphologically recognized sections are indicated within the clades. Outgroups are grey shaded.

**
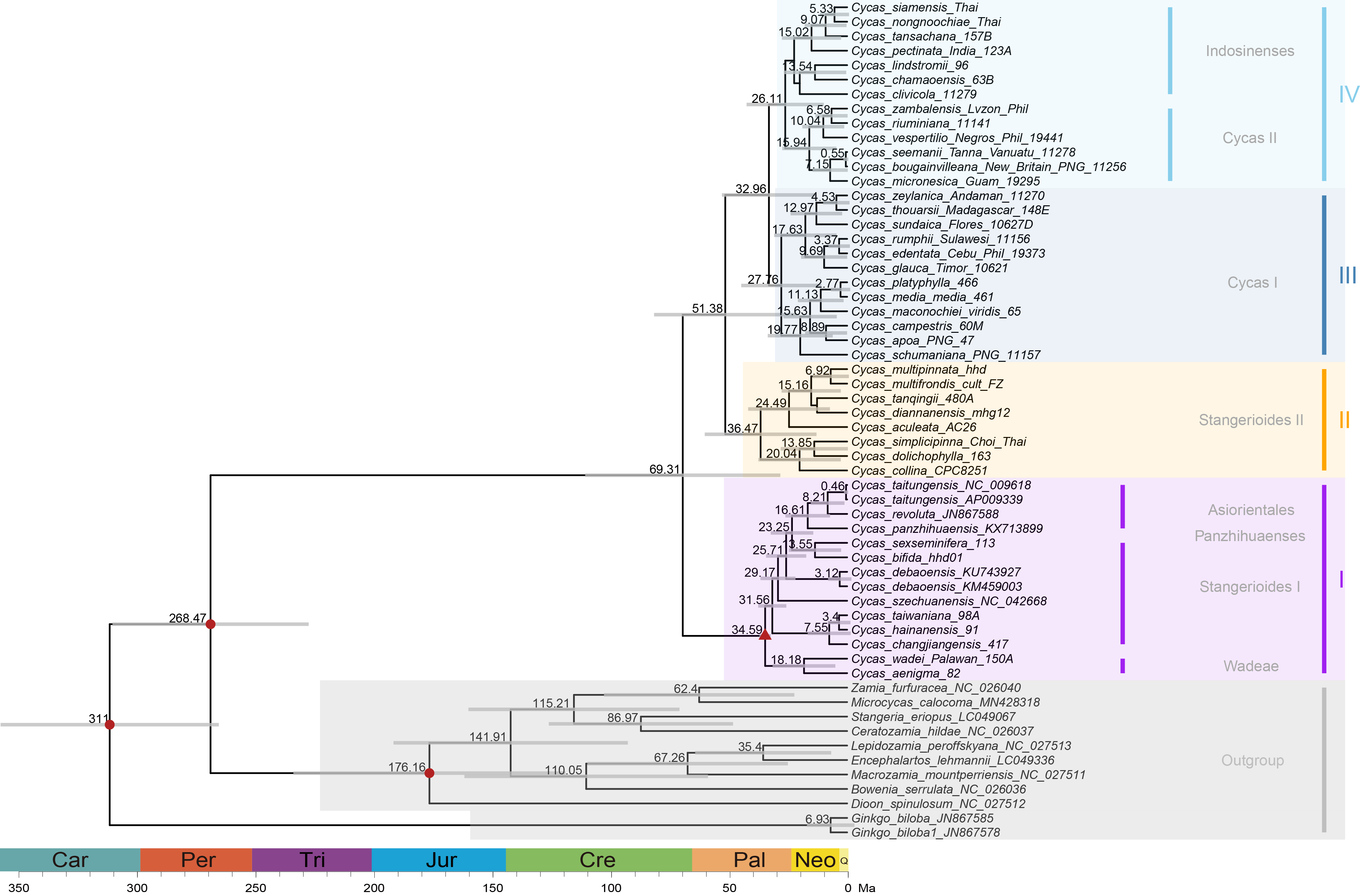
**

**Fig. S7.** Chronogram depicting the *Cycas* evolutionary timescale, as estimated by (a) birth-death and (b) Yule prior under calibration Scheme 3 based on 87 protein-coding genes using Bayesian analysis with an uncorrelated relaxed clock, in BEAST. Mean ages (in millions of years) are indicated for nodes, with grey bars indicating the 95% credibility intervals for these nodes. Morphologically recognized sections are indicated within the clades. Outgroups are grey shaded.

**
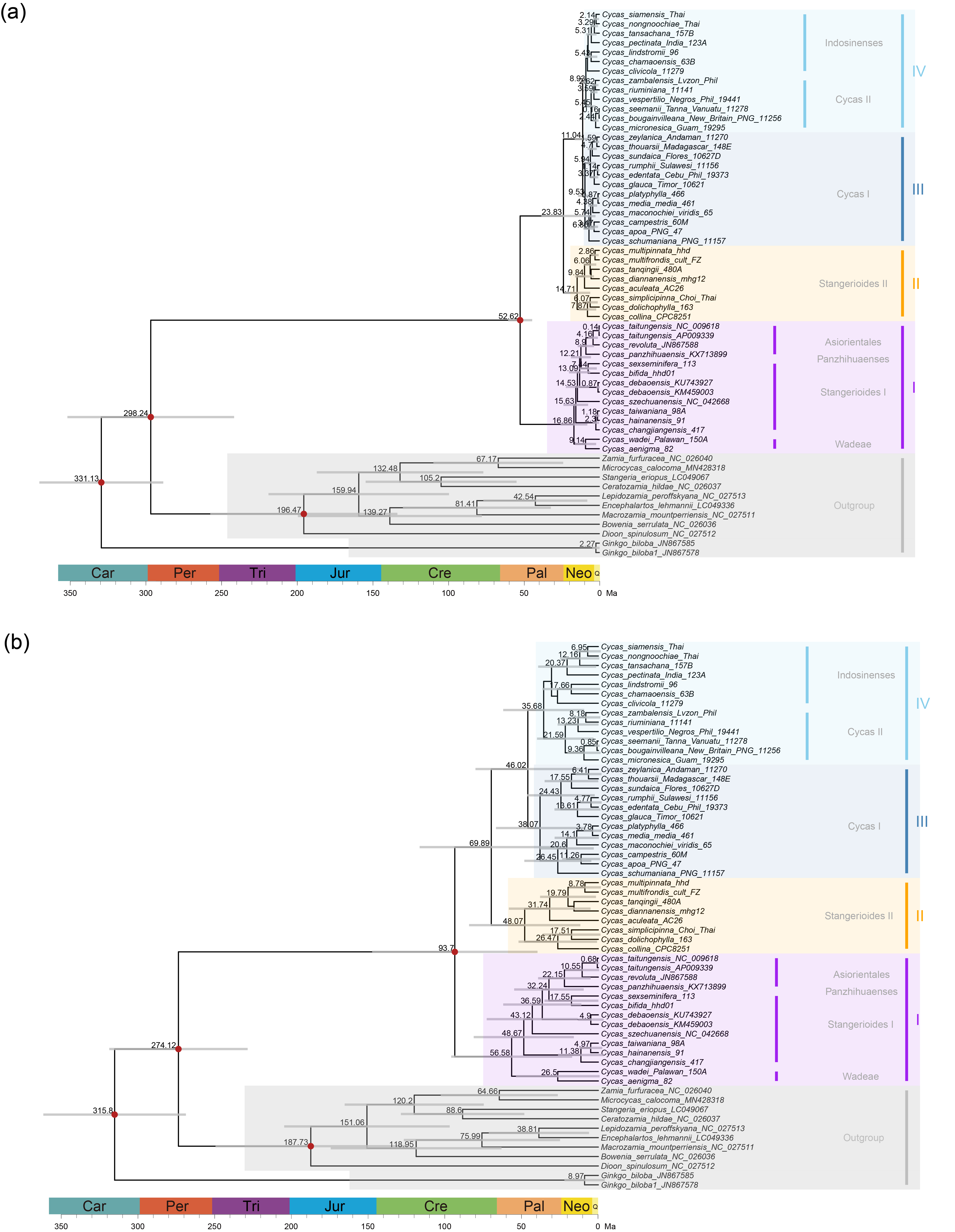
**

**Fig. S8.** Corrected (including the ratio of transitions to transversions) vs uncorrected pairwise differences for first and second codon positions (red), and third codon positions (purple) for the 87-gene dataset. The dashed line represents equal corrected and uncorrected pairwise differences. Note that the plot for third codon positions follows a linear relationship with increasing genetic distance, indicating no saturation.

**
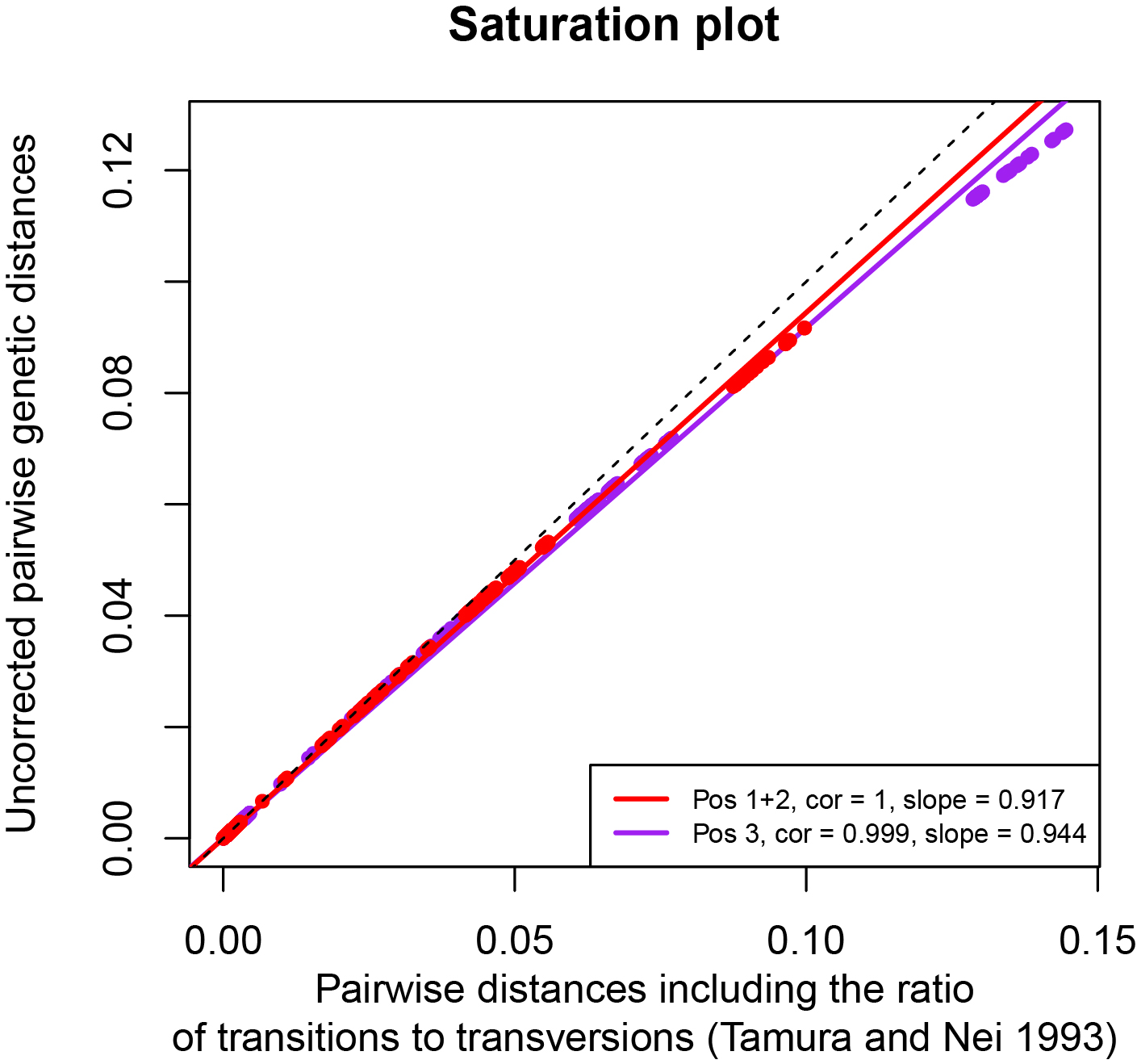
**

**Fig. S9.** Chronogram depicting the *Cycas* evolutionary timescale, as estimated by birth-death prior under calibration Scheme 1 based on the partitioned datasets with (a) the first and second positions of nucleotide codon, and (b) only the third positions of nucleotide codon, using Bayesian analysis with an uncorrelated relaxed clock, in BEAST. Mean ages (in millions of years) are indicated for nodes, with grey bars indicating the 95% credibility intervals for these nodes. Morphologically recognized sections are indicated within the clades. Outgroups are grey shaded.

**
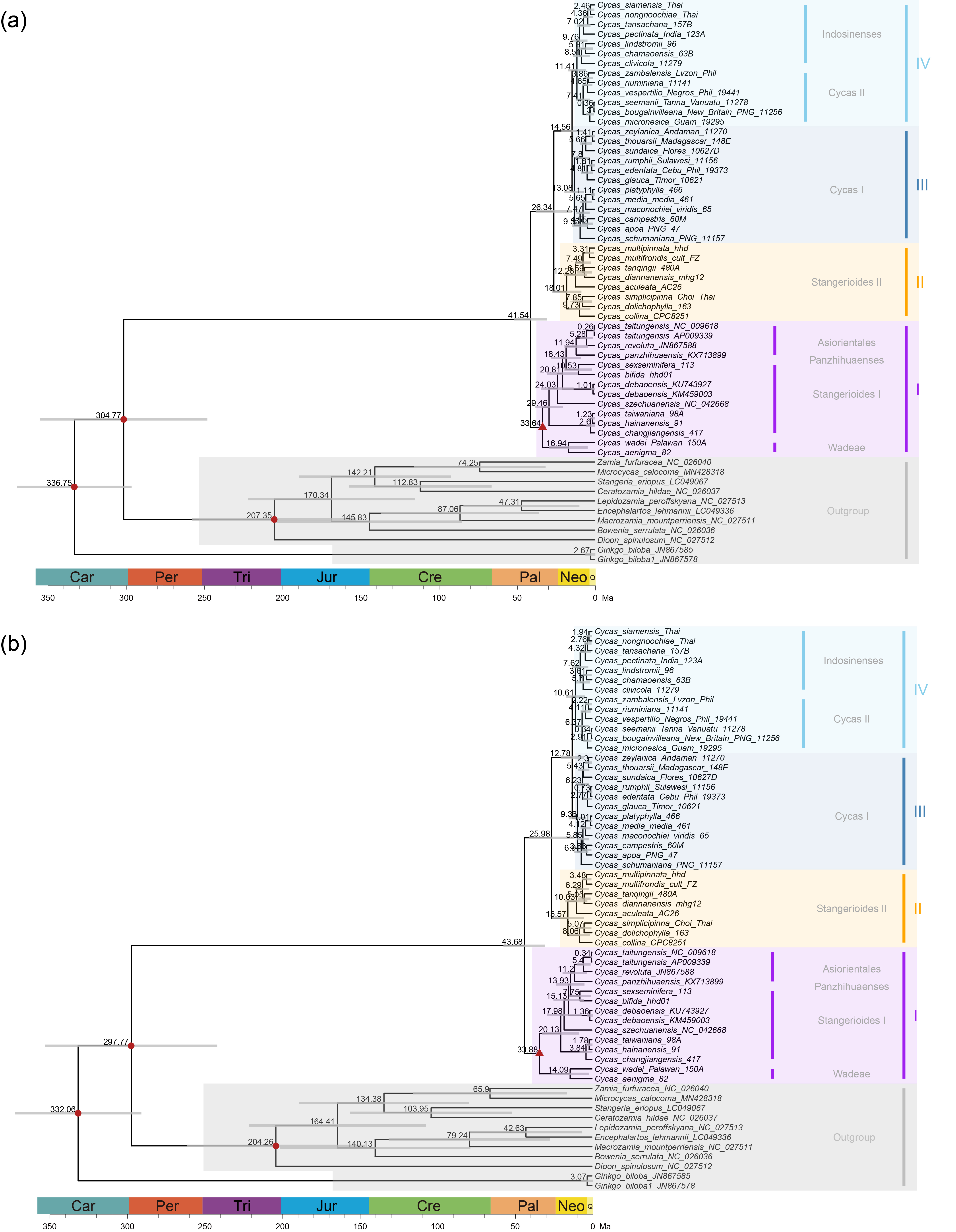
**

**Fig. S10.** Chronogram depicting the *Cycas* evolutionary timescale, as estimated by birth-death prior under calibration Scheme 1 based on a subsample of (a) 10, (b) 20, and (c) 40 protein-coding genes using Bayesian analysis with an uncorrelated relaxed clock, in BEAST. Mean ages (in millions of years) are indicated for nodes, with grey bars indicating the 95% credibility intervals for these nodes. Morphologically recognized sections are indicated within the clades. Outgroups are grey shaded.

**
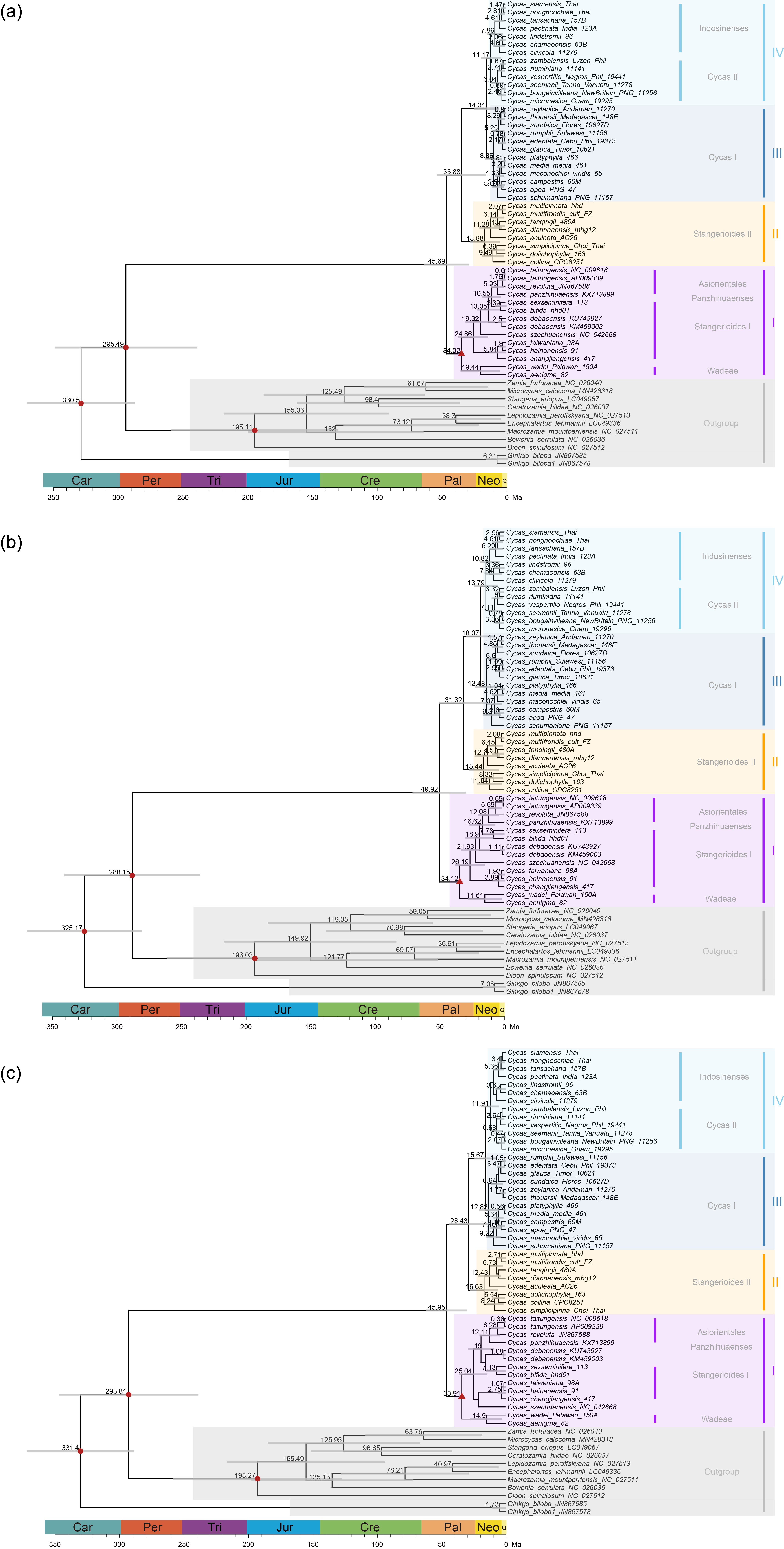
**

**Fig. S11.** Chronogram depicting the *Cycas* evolutionary timescale, as estimated by birth-death prior under calibration Scheme 1 based on 87 protein-coding genes using (a) strict clock, (b) random local clock, and (c) uncorrelated relaxed clock exponential model by Bayesian analysis in BEAST. Mean ages (in millions of years) are indicated for nodes, with grey bars indicating the 95% credibility intervals for these nodes. Morphologically recognized sections are indicated within the clades. Outgroups are grey shaded.


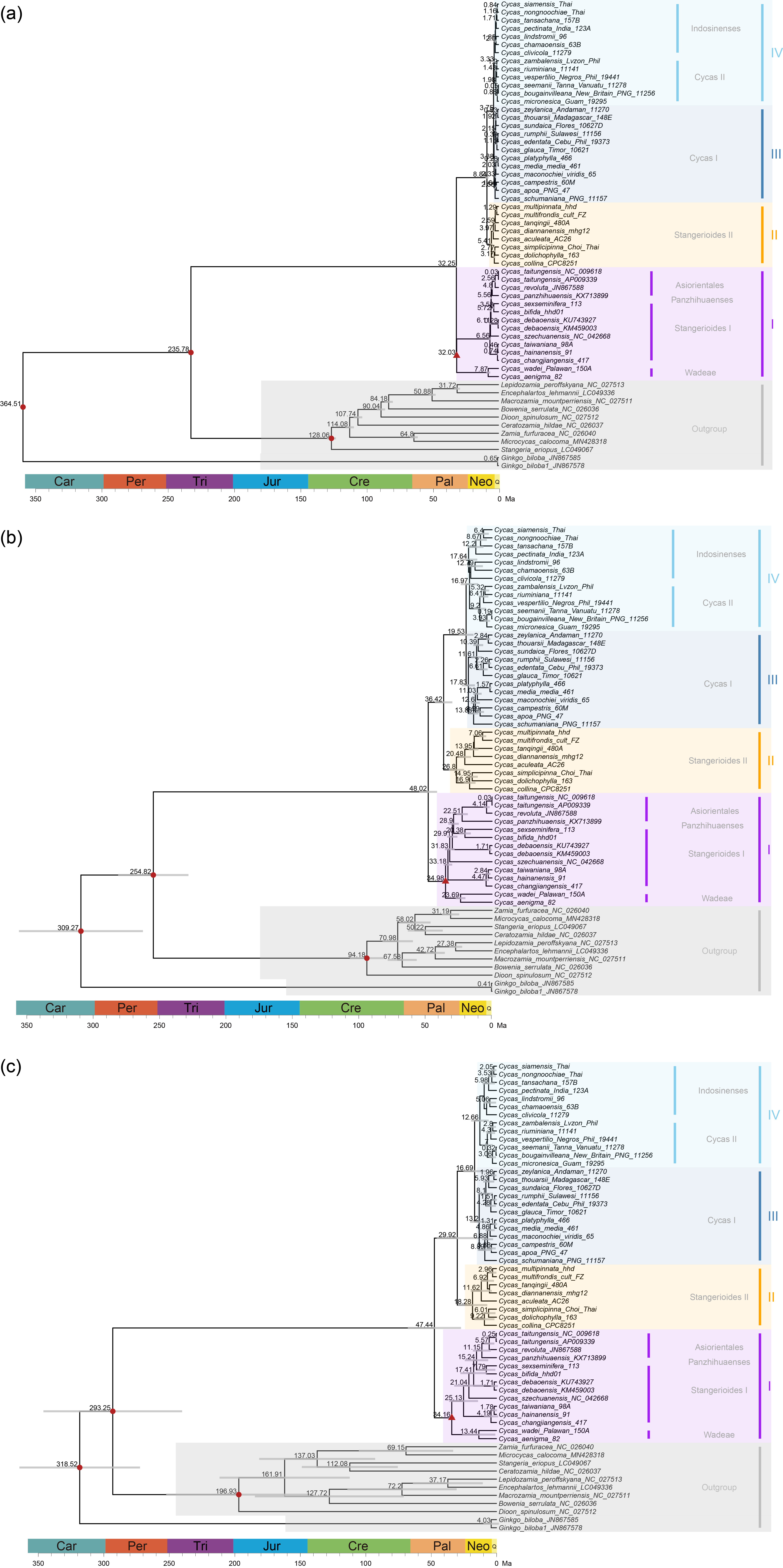


**Fig. S12.** Biogeographic reconstruction based on Statistical-Dispersal-Extinction-Cladogenesis (S-DEC) method and distributions of extant *Cycas*. The tree is summarized from 1000 randomly sampled BEAST posterior trees by RASP. Colored dots at nodes represent most likely ancestral areas. Inset is the geographic distribution of extant Cycadaceae (*Cycas*), including the five geographic regions used: A: East Asia, south to the Red River region, B: Indochina and India, C: Palawan and Culion islands, D: Southeast Asia islands including Australia, E: Africa.


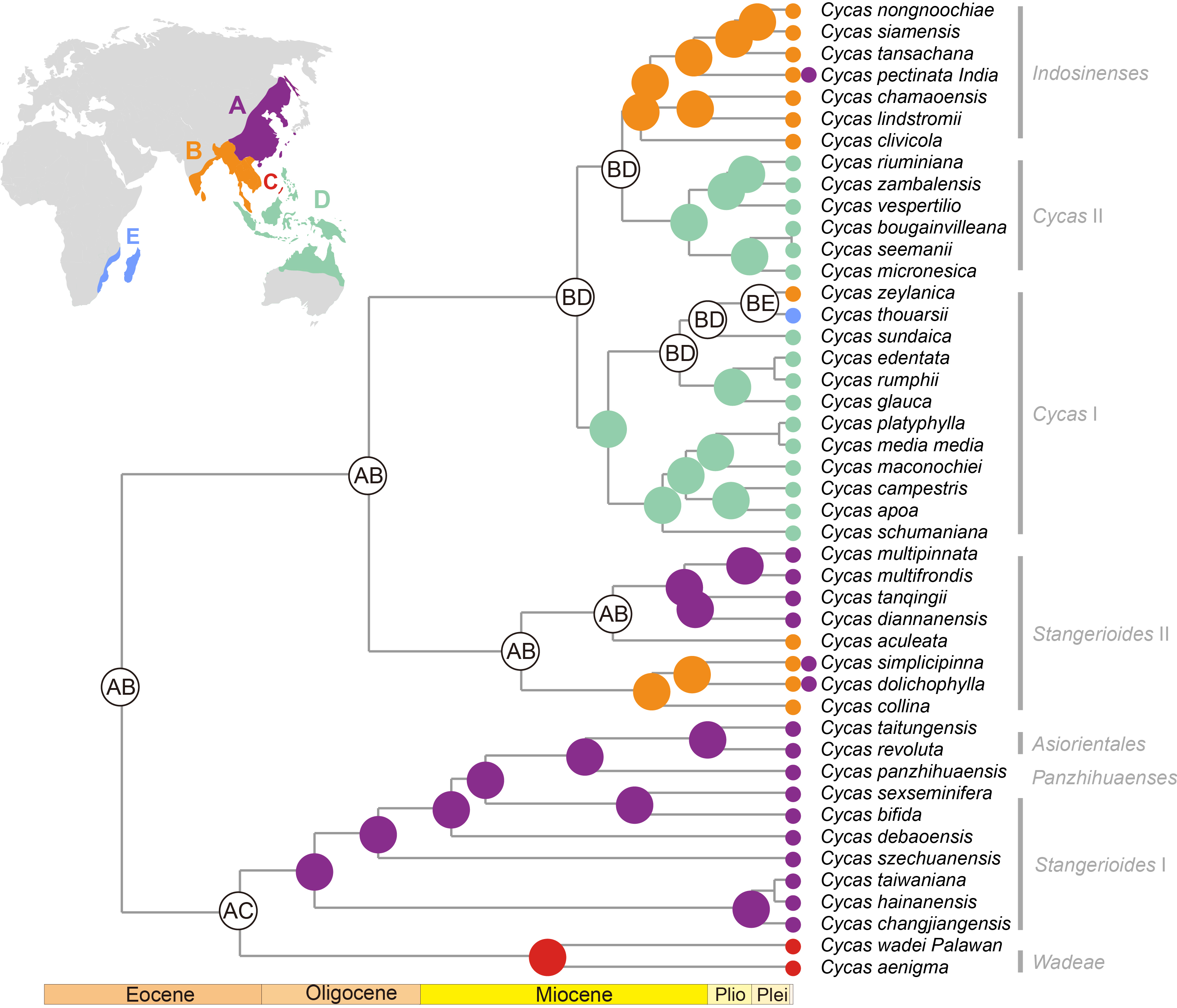

Supplement: mcab118_suppl_Supplementary_Materials_S2 [file mcab118_suppl_supplementary_materials_s2.docx]
